# Brain structural associations with depression in a large early adolescent sample (the ABCD study®)

**DOI:** 10.1016/j.eclinm.2021.101204

**Published:** 2021-11-20

**Authors:** Xueyi Shen, Niamh MacSweeney, Stella W.Y. Chan, Miruna C. Barbu, Mark J. Adams, Stephen M. Lawrie, Liana Romaniuk, Andrew M. McIntosh, Heather C. Whalley

**Affiliations:** a Division of Psychiatry, University of Edinburgh, Royal Edinburgh Hospital, Morningside Park, Edinburgh EH10 5HF, United Kingdom; b Department of Clinical Psychology, University of Edinburgh, Edinburgh, United Kingdom

**Keywords:** Big data, Adolescent depression, Adolescent Brain and Cognitive Development Study, Brain structure

## Abstract

**Background:**

Depression is the leading cause of disability worldwide with > 50% of cases emerging before the age of 25 years. Large-scale neuroimaging studies in depression implicate robust structural brain differences in the disorder. However, most studies have been conducted in adults and therefore, the temporal origins of depression-related imaging features remain largely unknown. This has important implications for understanding aetiology and informing timings of potential intervention.

**Methods:**

Here, we examine associations between brain structure (cortical metrics and white matter microstructural integrity) and depression ratings (from caregiver and child), in a large sample (*N* = 8634) of early adolescents (9 to 11 years old) from the US-based, Adolescent Brain and Cognitive Development (ABCD) Study**®**. Data was collected from 2016 to 2018.

**Findings:**

We report significantly decreased global cortical and white matter metrics, and regionally in frontal, limbic and temporal areas in adolescent depression (Cohen's *d* = -0⋅018 to -0⋅041, β = -0·019 to -0⋅057). Further, we report consistently stronger imaging associations for caregiver-reported compared to child-reported depression ratings. Divergences between reports (caregiver vs child) were found to significantly relate to negative socio-environmental factors (e.g., family conflict, absolute β = 0⋅048 to 0⋅169).

**Interpretation:**

Depression ratings in early adolescence were associated with similar imaging findings to those seen in adult depression samples, suggesting neuroanatomical abnormalities may be present early in the disease course, arguing for the importance of early intervention. Associations between socio-environmental factors and reporter discrepancy warrant further consideration, both in the wider context of the assessment of adolescent psychopathology, and in relation to their role in aetiology.

**Funding:**

Wellcome Trust (References: 104036/Z/14/Z and 220857/Z/20/Z) and the Medical Research Council (MRC, Reference: MC_PC_17209).

**Research in context**

**Evidence before this study**

While disruptions in brain structure have been associated with depression in adult samples, less is known about the temporal origin and development of these depression-related imaging features. We searched Google Scholar for studies published in English from inception to 2021, using search terms “MDD OR major depression OR major depressive disorder OR depression OR depress(ive/ion) symptoms” “AND adolescent OR adolescence” “AND brain structure OR cortical OR subcortical OR white matter OR MRI”. We did not find any studies that used multi-modal imaging data from large, population samples to investigate brain structural associations with depression in adolescence.

**Added value of this study**

To our knowledge, this is the first study to use the Adolescent Brain and Cognitive Development (ABCD) Study® to examine cortical and white matter microstructural associations with adolescent depression. We report shared depression-related imaging features between adolescent and adult literature, such as reduced white matter integrity in frontal-limbic circuits. Our findings also demonstrate surface area differences in this sample, which may represent an adolescent-specific vulnerability.

**Implications of all the available evidence**

Our findings suggest that cortical and white matter microstructural abnormalities exist early in the disease course of depression. Tracing the roots of depression in the developing brain provides opportunity to better our understanding of the factors associated with its emergence during this period of neurodevelopmental sensitivity. However, longitudinal data, which will soon be available through the ABCD Study, is needed to chart intra- and inter-personal neurodevelopmental trajectories to identify modifiable risk factors that could inform future intervention approaches.

Alt-text: Unlabelled box

## 1 Introduction

Major depressive disorder (MDD) is a chief cause of disability [1] with a heritability of approximately 37% [2]. This burden falls heavily on adolescents, as over 50% of depression cases emerge before the age of 25 [3]. Adolescent depression is notably associated with a more severe illness course and can lead to the propagation of difficulties across the lifespan [4]. MDD is associated with disruptions in brain structure [5], [6], [7], [8]. However, due to a lack of large-scale neuroimaging samples for adolescents, the origin and development of depression-related imaging features remains largely unknown.

Large population-based neuroimaging studies in adults have allowed unparalleled insight into the neurobiological underpinnings of depression [5,6]. For example, recent evidence from the ENIGMA (Enhancing NeuroImaging Genetics through Meta-Analysis) consortium demonstrated widespread structural abnormalities in MDD from large adult samples, including reduced hippocampal volume, decreased frontal cortical thickness [8] (*N* = 10,105) and altered fronto-limbic and fronto-thalamic tract microstructure [9] (*N* = 2907). Since these highly powered studies have largely been conducted in adults, they preclude investigation of the neurobiology underlying the emergence and development of depression earlier in life. Given adolescence is the period of greatest risk for the development of depression [4], as well as a time of immense neurodevelopmental change [10,11], it is a key period in which to investigate evidence for emergence of these imaging features.

Findings from earlier studies on brain structural alterations in adolescent MDD have been highly heterogeneous [12], [13], [14], [15], [16], [17]. A recent meta-analysis of imaging studies of MDD from ENIGMA, which included a relatively large adolescent population (*N* = 507, age range 12–21 years), indicated lower global surface area and regional reductions in frontal areas in this younger sample of depressed cases [8]. However, this subsample comprised primarily of participants from older adolescence to young adulthood, where 90% of the sample were aged ≥ 16 years, meaning earlier origins of depression related brain imaging features remain under explored. There have also been recent efforts to investigate whether white matter integrity disruptions seen in adult cases are present in adolescent depression. Although some studies report reduced white matter microstructural integrity in adolescents with depression, findings have lacked consistency in terms of regions [18], [19], [20] and effects sizes [21,22], likely due to small sample sizes [18], [19], [20]. Moreover, symptom heterogeneity may contribute to these disparate findings as reduced white matter integrity has been found to relate to depression sub-types [23] as well as subthreshold depression [24]. It therefore remains unclear whether reduced white matter integrity is a hallmark of early depression pathophysiology during adolescence.

There is a significant degree of individual difference in symptom presentation and impairment in adolescents experiencing depressive symptoms, especially regarding the social context in which these difficulties manifest (e.g., home, school). Discordance between child and parent reports of psychopathology has been well documented [25,26] with some research suggesting that parents may under-report youth depressive symptoms compared to youth self-report [27]. However, given inconsistent findings in the literature [28], a multiple-informant approach, which usually includes the young person and their caregiver, remains “best-practice” [29,30]. Notably, the associated implications of reporter discrepancy in youth psychopathology [26] has been understudied in the context of underlying neurobiological associations. We extend this existing work by looking at both caregiver and child reported symptoms and how these differentially associate with imaging features.

The current study therefore examines early associations between multi-modal structural imaging features and the emergence of MDD and depressive symptoms (DS) from the population-based, demographically diverse, Adolescent Brain and Cognitive Development (ABCD) Study**®**, using both caregiver and child reported symptoms [31]. The ABCD Study® is a population-based longitudinal project that encompasses magnetic resonance imaging (MRI) data and lifetime assessments of psychiatric disorders in 9–11-year-old US children (*N*  = 8634, mean age = 9⋅91 years).

## 2 Methods

### 2.1 Participants

Data from the curated annual release 2.0.1 of the Adolescent Brain Cognitive Development (ABCD) Study® were used. Participants were recruited from 21 sites across the United States [32]. A total of *N* = ~11,800  children (9–11 years) participated in the baseline assessment, which took place between September 1st 2016 and August 31st 2018. The unrelated participants with quality-controlled brain imaging measures (cortical measures or white matter measures) were included in the analysis (*N* = 8634, mean age = 9⋅91, standard deviation = 0⋅62, 52⋅3% were male). The study was approved by the National Institute of Mental Health Data Archive, United States (NIMH). Written consent was obtained from all participants. Data was accessed through the NDA data base (https://nda.nih.gov/general-query.html?q=query=featured-datasets:Adolescent%20Brain%20Cognitive%20Development%20Study%20(ABCD); Federal-Wide Assurance: FWA00018101). Further details can be found in Table 1. Demographic information for those with missing data can be found in Table S1.

**Table 1:** Sample sizes and demographic features for MDD and depressive symptoms (DS).

			N	Age		Sex (% of Male)
				Mean	SD	
Total sample			8634	9.91	0.62	52.3%
						
MDD	Reported by caregivers	Case	194	10.02	0.6	53.6%
		Control	6683	9.89	0.62	51.6%
	Reported by children	Case	180	9.95	0.63	58.9%
		Control	6744	9.9	0.62	51.4%
						
DS	Reported by caregivers	Severe	60	10.1	0.56	51.7%
		Moderate	386	9.91	0.61	54.1%
		Mild	208	9.96	0.62	58.2%
		None of the above	7980	9.91	0.62	52.1%
	Reported by children	Severe	59	10	0.61	61.0%
		Moderate	423	9.91	0.62	53.9%
		Mild	205	9.86	0.6	52.7%
		None of the above	7926	9.91	0.62	52.1%

### 2.2 Derived brain structural measures

Brain imaging data were acquired and processed by the ABCD team. A 3-T Siemens Prisma, General Electric 750 or Phillips scanner was used for data acquisition. A unified protocol for the scanning was used to harmonise between sites and scanners. Protocols used for data acquisition and processing were described elsewhere [31]. Standard preprocessing and quality check (QC) procedures were conducted according to the ABCD protocol. Participants with excessive head motion or poor data quality were excluded from the curated data release.

Two types of brain structural measures were used in the present study: grey matter cortical and white matter microstructural measures.

Cortical measures were generated using Freesurfer 5.3.0 (https://surfer.nmr.mgh.harvard.edu/fswiki/FreeSurferWiki). Four types of cortical measures were used: surface area, thickness, volume and sulcal depth. First, global measures were generated for each cortical measure over the whole brain (see Fig. S1). The Desikan-Killiany atlas was then used for parcellation of 34 bilateral regional cortical structures.

White matter microstructural measures included fractional anisotropy (FA) and mean diffusivity (MD). Global measures of FA and MD were generated over the whole brain. The TractAtlas was used to map boundaries of the 14 bilateral and 3 unilateral major tracts [33]. FA/MD values were then derived for each of the region.

Data with poor-quality raw T1/DTI scans and low post-processing QC scores were removed. As there were outlying values for white matter microstructural measures, we removed those with global FA and MD values 5 standard deviations from mean [5]. Further details can be found in Supplementary Information.

### 2.3 Measures for major depressive disorder and depressive symptoms in adolescents

Life-time Major Depressive Disorder (MDD) and depressive symptoms (DS) for children were assessed using a computerised version of the Kiddie Schedule for Affective Disorders and Schizophrenia (KSADS) [34]. The scale included 28 binary items on current and past DS that reached clinical significance [34] (Table S2). Questions were completed by parents and children separately and self-administered. A previous study of the computerised version for scales showed good to high reliability, with AUC = 0.89–1.00 comparing against clinician administered, computerised KSADS diagnoses [35]. Lifetime measures of MDD and DS were generated by combining reports on current and past symptoms (a positive answer for either current or past were grouped as positive for lifetime depression, and negative answers on both were grouped as negative, see Supplementary Methods). Questionnaires were completed by both children and by a caregiver independently. A diagnosis of MDD was generated by the ABCD team for both child and caregiver reported symptoms separately [36]. We additionally created a measure of DS based on Diagnostic and Statistical Manual of Mental Disorders (DSM-V) criteria for the severity scale of depression [37]. Levels of DS included: ‘severe’, ‘moderate’, ‘mild’ and ‘none of the above’ (encoded as 3–0, respectively, see Tables S2 and 3, Fig. S4 and Supplementary Methods). DS assessed using the Child Behaviour Checklist (CBCL, a Likert-scale measure) based on reports by caregivers were also used to validate these measures [38] (see Supplementary Information). MDD and DS for unrelated children were included in the analysis. Sample sizes for these variables can be found in Table 1.

In addition to the DS reported by caregivers and children separately, we looked at the average reports and discrepancies of DS. Average DS was obtained by calculating the mean of DS reported by each caregiver-child pair. Discrepancy was generated by obtaining the absolute values of subtracting caregiver and child reports.

We also sought to control for potential biases introduced by the current mood of caregivers which could confound associations between their rating of depression in the adolescents with the brain structural measures. We used a subscale of DSM-V-oriented items for depressive problems from the Adult Self-Report (ASR) in the Achenbach System of Empirically Based Assessment [36] (Supplementary Methods).

### 2.4 Measures of socio-environmental factors

As exploratory analyses to further understand potential socio-environmental factors relating to differences in caregiver and child report, we also examined absolute discrepancies in these reports with variables from the ABCD sample including cultural, social, family and school environment of children reported by both caregivers and children themselves, see Supplementary Methods and Table S4.

### 2.5 Statistical models

Statistical analyses were performed in Scientific Linux 2.6.32, using R 3.6.1.

Firstly, associations between MDD diagnosis (binary) and number of depressive items reported DS (ordinal) in adolescents and brain structural measures were tested using a General Linear Model (GLM, ‘glm’ function) or Linear Mixed-effect model (LME, ‘lme’ function) [39] in R. For unilateral brain measures, a GLM was used. For bilateral brain measures, an LME model was used with hemisphere set as a repeated measure [5]. Covariates included age, age^2^, sex, ethnicity, study site, recent social deprivation and additional imaging covariates: head motion (data field: ‘fsqc_qu_motion’) and hemisphere for the LME models (see Table S5). To further test if current caregivers’ mood confounded associations between depression in adolescents and brain structural measures, we added ASR scores of caregivers as a covariate and compared the results with the main model.

The analyses of associations with brain structural measures followed a hierarchical order from global measures at the whole-brain level to individual structures. For cortical measures, this included whole brain cortical volume, mean thickness, total surface area, mean sulcal depth, followed by individual brain regions. For white matter microstructural measures (FA/MD), the global ‘g’ measures were first tested, followed by individual tracts. The *p* values were corrected using family-wise error correction with the FDR (false discovery rate) method [40], using the ‘p.adjust’ function in *R*. This was applied for each brain measure category and each reporter separately.

In addition to the main models, we conducted analyses on the mean and discrepancy of DS reported by caregivers and children. Average reports and discrepancies for DS was generated for each child-caregiver pair. Results for the associations between the average severity and general/regional brain measures are shown in the Supplementary Information.

We conducted sensitivity analyses to test potential confounding effects of MRI sites, scanner manufacturers and anti-depressant use in the adolescents (methods and results reported in the Supplementary Information).

To examine origins of the discrepancy of report, we tested which socio-environmental factors were associated with the discrepancy. *R* function ‘kappa2’ from package ‘irr’ was used to estimate Cohen's Kappa for testing agreement between caregiver and child reports (https://www.rdocumentation.org/packages/irr/versions/0.84.1). GLM models were used and covariates kept consistent with models above with the exception of removing imaging covariates (methods reported in Supplementary Information). As these measures were more likely to be independent tests rather than correlated (e.g. brain structural measures), we applied Bonferroni-correction [41].

The current study adheres to the STROBE reporting guidelines. Data used in the preparation of this article were obtained from the Adolescent Brain Cognitive Development (ABCD) Study® (https://abcdstudy.org), held in the NIMH Data Archive (NDA). The ABCD Study® is supported by the National Institutes of Health and additional federal partners under award numbers U01DA041048, U01DA050989, U01DA051016, U01DA041022, U01DA051018, U01DA051037, U01DA050987, U01DA041174, U01DA041106, U01DA041117, U01DA041028, U01DA041134, U01DA050988, U01DA051039, U01DA041156, U01DA041025, U01DA041120, U01DA051038, U01DA041148, U01DA041093, U01DA041089, U24DA041123, U24DA041147. A full list of supporters is available at https://abcdstudy.org/federal-partners.html. A listing of participating sites and a complete listing of the study investigators can be found at https://abcdstudy.org/consortium_members/. ABCD consortium investigators designed and implemented the study and/or provided data but did not necessarily participate in the analysis or writing of this report. This manuscript reflects the views of the authors and may not reflect the opinions or views of the NIH or ABCD consortium investigators. The ABCD data repository grows and changes over time. The ABCD data used in this report came from NDA Study ID: 1260; DOI: 10.15154/1522853.

### 2.6 Role of funding sources

Our funding sources (Wellcome Trust and Mental Health Research UK) were not involved in the study preparation/design, analysis/interpretation of data, or in the writing and submission of this report.

## 3 Results

### 3.1 Major depressive disorder (MDD), depressive symptoms (DS) and brain measures

Description of individuals meeting criteria for MDD and reporting DS at mild level and above by both parent (MDD: *N* = 194 (2.82%), DS: *N* = 654 (7.57%) and child (MDD: *N* = 180 (2.60%), DS: *N* = 687 (7.98%)) are reported in Table 1. For caregiver report, youth who had DS of mild, moderate and severe type were 0.69% (*N* = 60), 4.47% (*N* = 386), and 2.41% (*N* = 208), respectively. For child self-report, individuals reporting mild, moderate and severe DS were 0.69% (*N* = 59), 4.91% *(N* = 423), and 2.38% (*N* = 205), respectively.

### 3.2 Global imaging metrics

**Fig. 1:**
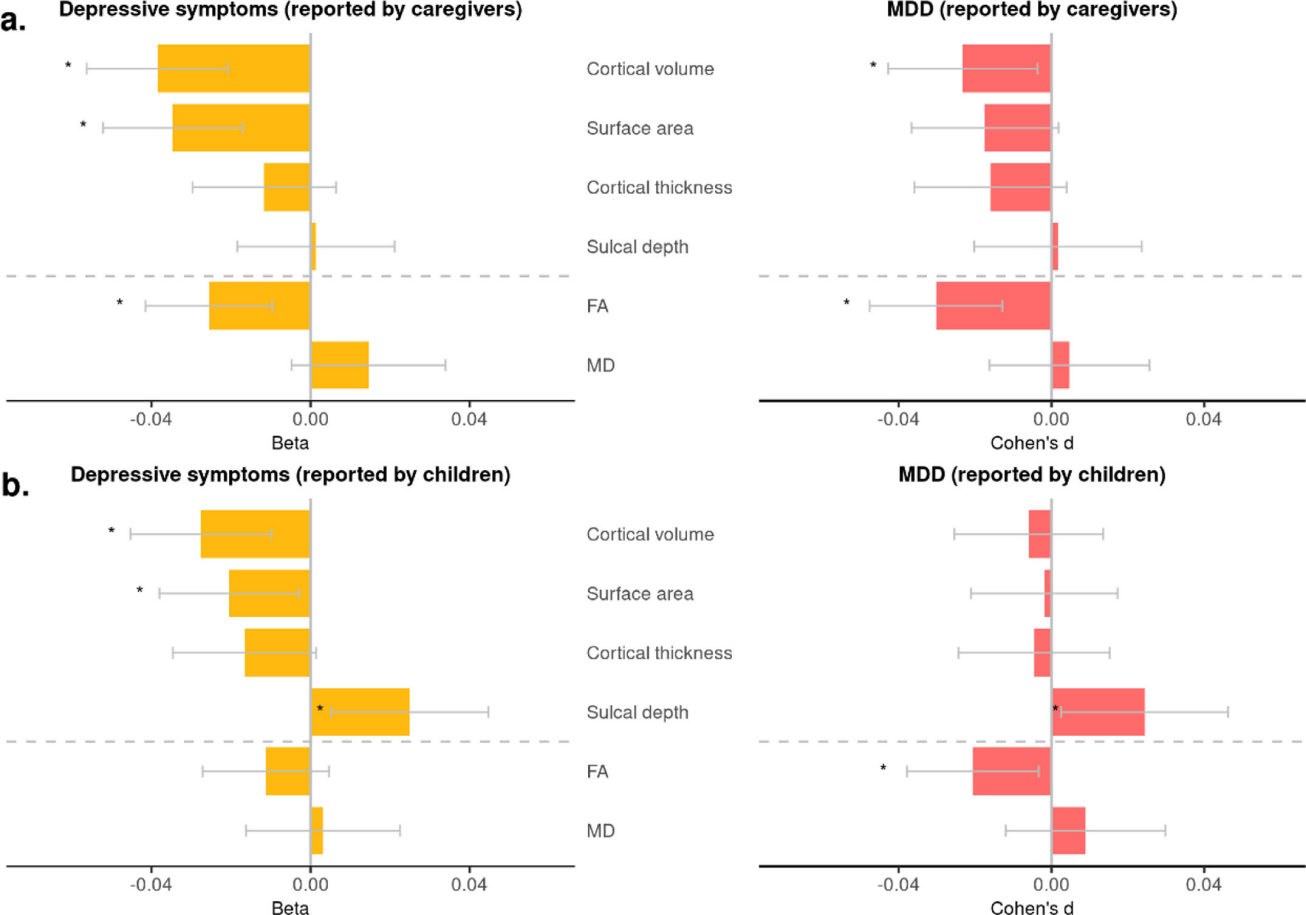
Associations between Major depressive disorder (MDD), depressive symptoms (DS) and general measures of cortical and white-matter structures. X-axes represent standardised effect sizes, and *y*-axes represent each general measure of brain structure. Error bars represent the 95% confidence interval. Panel (a) shows the results for MDD/depressive symptoms reported by caregivers on children, and panel (b) shows the results for MDD/depressive symptoms reported by children themselves.

*Caregiver report:* Global results are shown in Fig. 1 and Supplementary Data 1. MDD diagnosis for the child as reported by caregivers was associated with significantly lower total cortical volume (Cohen's *d* = −0⋅022, *p* = 0⋅013) and global FA (Cohen's *d* = −0⋅027, *p* = 7⋅96 × 10^−4^). Increasing summary measures of DS reported by caregivers were associated with decreased total cortical volume (β = −0⋅037, *p* = 5⋅18 × 10^−5^), total surface area (β = −0⋅034, *p* = 1⋅41 × 10^−4^) and global FA (β = −0⋅023, *p* = 4⋅06 × 10^−3^).

*Child report:* Associations between imaging measures and the child reported measures are also shown in Fig. 1. In general, the associations were weaker than the above reports by caregivers, with fewer significant associations (see Fig. 1 and Supplementary Data 1). MDD diagnosis based on child report was significantly associated with increased cortical sulcal depth (Cohen's *d* = 0⋅020, *p* = 0⋅040) and lower global FA (Cohen's *d* = −0⋅018, *p* = 0⋅022), and DS were associated with lower total cortical volume (β = −0⋅027, *p* = 2⋅93 × 10^−3^), smaller total surface area (β = −0⋅020, *p* = 0⋅022) and greater sulcal depth (β = 0⋅023, *p* = 0⋅023).

### 3.3 Regional brain metrics

**Fig. 2:**
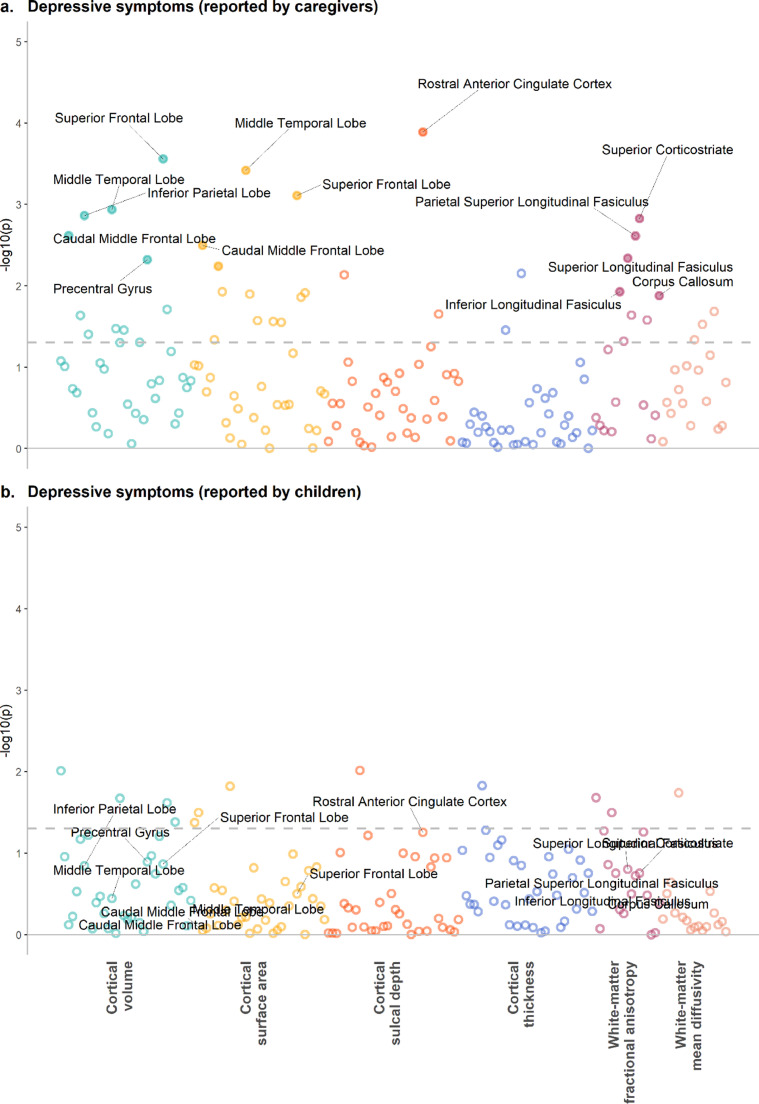

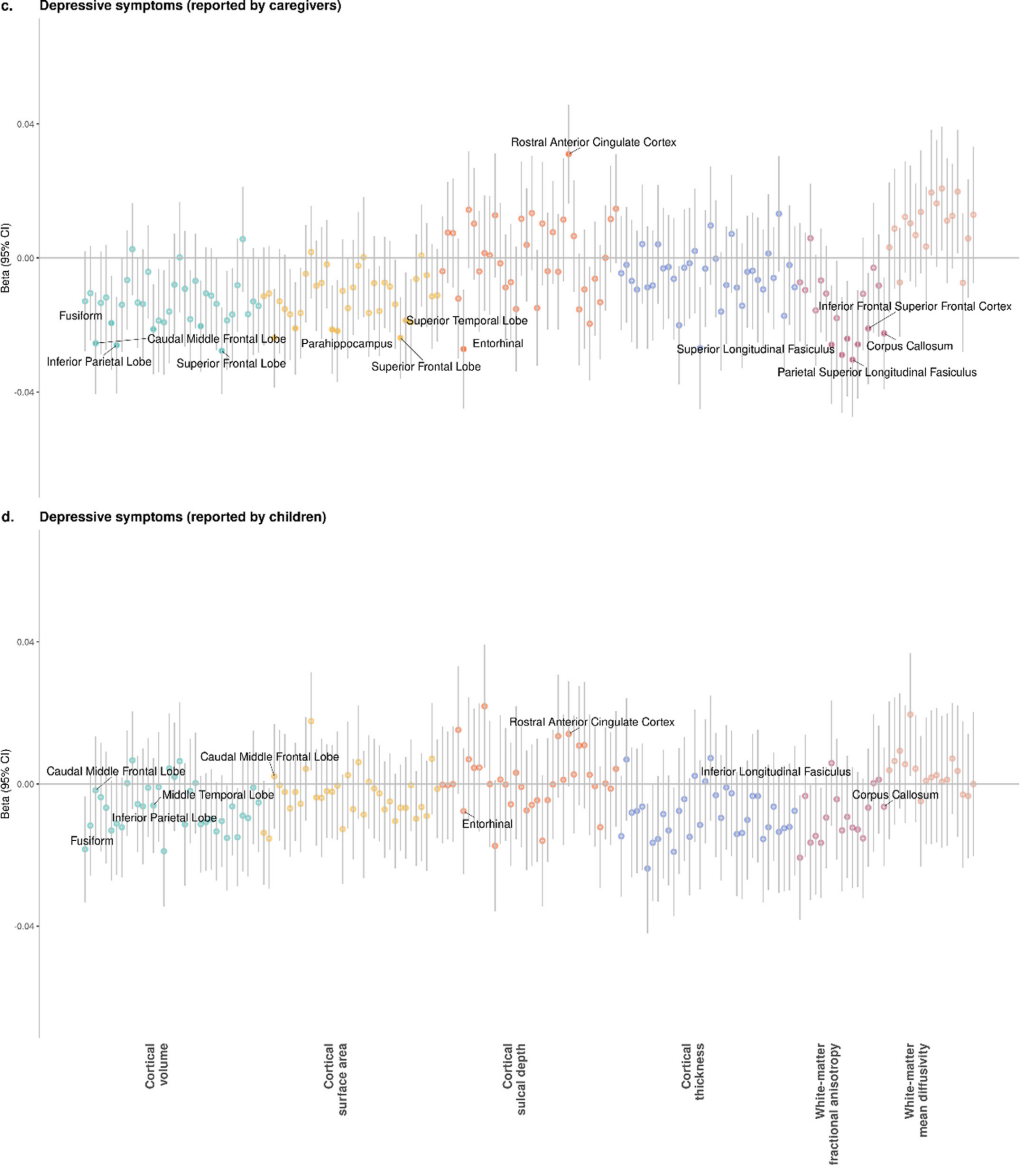
*P*-value plots for associations between depressive symptoms and measures for regional brain regions. X axes represent individual brain structural measures, and *y* axes represent -log10 transformed p-values. Panels (a) and (b) present the *p*-value statistics for depressive symptoms reported by caregivers on children and for symptoms reported by children themselves, respective. Panels (c) and (d) show the standardised regression coefficients and 95% confidence intervals for depressive symptoms reported by caregivers on children and for symptoms reported by children themselves, respective. Solid dots represent variables significantly associated with depressive symptoms after FDR-correction. For clarity, threshold for nominal significance before FDR-correction is shown as the grey dashed line in panels (a) and (b).

**Fig. 3:**
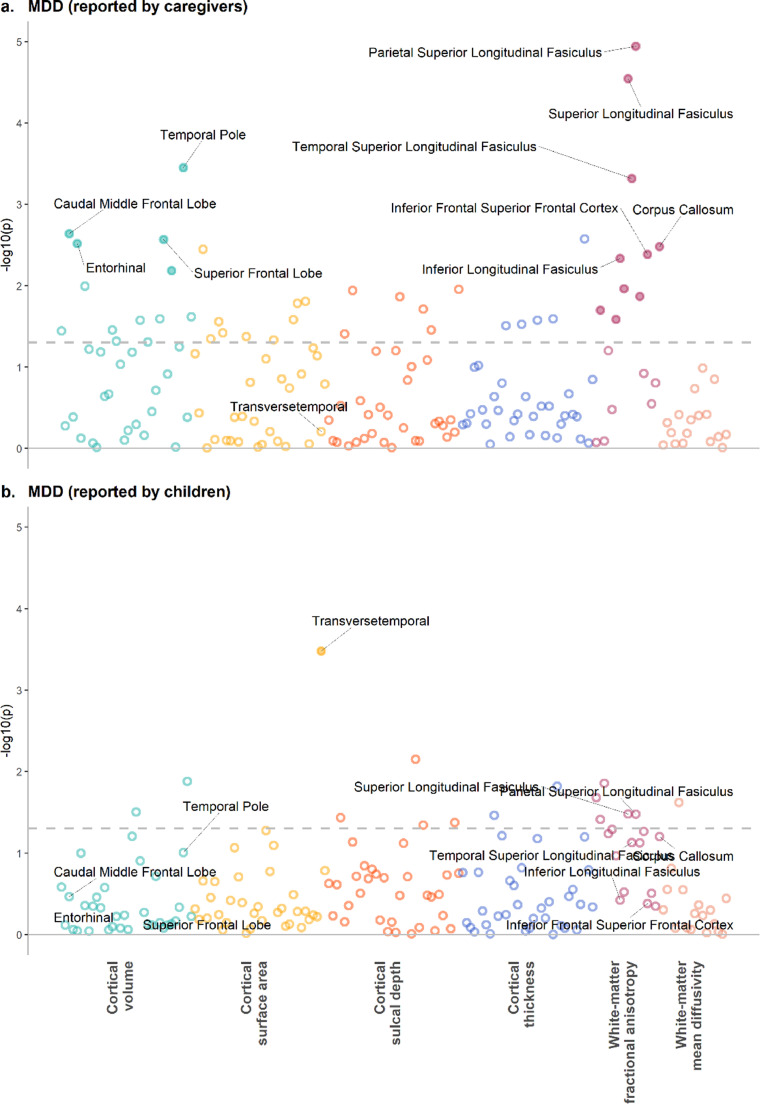

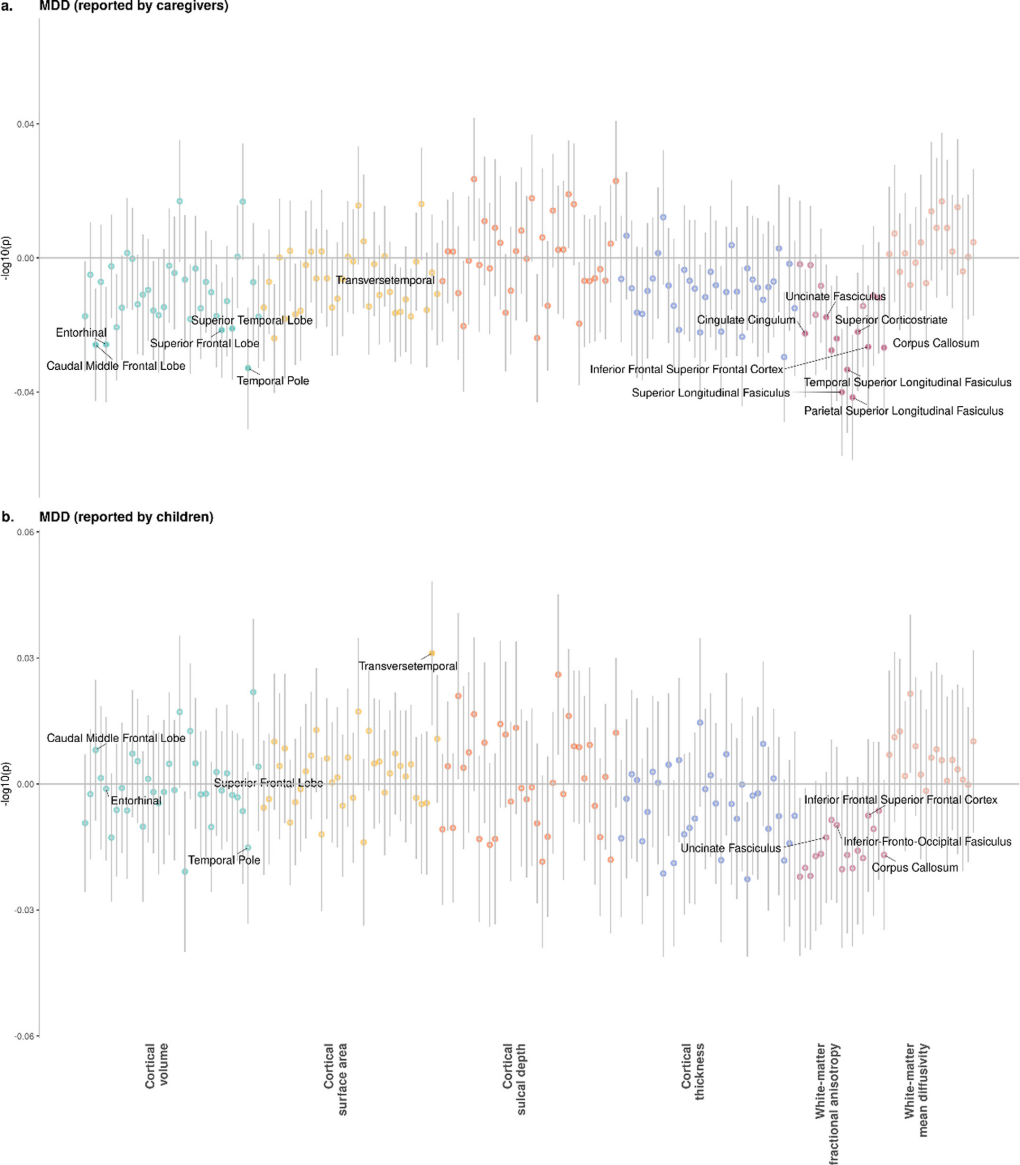
*P*-value plots for associations between MDD and measures for single brain regions. X axes represent individual brain structural measures, and y axes represent -log10 transformed *p*-values. Panels (a) and (b) present the *p*-value statistics for MDD reported by caregivers on children and for symptoms reported by children themselves, respective. Panels (c) and (d) show the standardised regression coefficients and 95% confidence intervals for MDD reported by caregivers on children and for symptoms reported by children themselves, respective. Solid dots represent variables significantly associated with depressive symptoms after FDR-correction. For clarity, threshold for nominal significance before FDR-correction is shown as the grey dashed line in panels (a) and (b).

*Caregiver report:* Regional results are shown in Figs. 2 and 3 and Supplementary Data 2–5. For cortical measures, MDD diagnosis-based reports by caregivers was associated with reduced volumes of the caudal middle frontal lobe, entorhinal cortex, superior frontal lobe, superior temporal lobe, and temporal pole (Cohen's d range: −0⋅019 to −0⋅029, p_FDR_ range: 0⋅043 to 0⋅012). Volumes in caudal middle frontal lobe and superior frontal lobe were also associated with DS, along with volumes in other regions that include inferior parietal lobe, middle temporal lobe and precentral gyrus (β range: −0⋅019 to −0⋅024, p_FDR_ range: 0⋅041 to 0⋅012). Smaller surface area of similar regions was associated with higher DS, which include caudal middle frontal lobe, inferior parietal lobe, middle temporal lobe and superior frontal lobe (β range: −0⋅020 to −0⋅024, p_FDR_ range: 0⋅049 to 0⋅013). Sulcal depth of rostral anterior cingulate was also associated with higher DS (β = 0⋅029, p_FDR_ = 0⋅004).

For white matter microstructural measures, MDD diagnosis by caregivers was associated with lower FA in uncinate fasciculus, inferior longitudinal fasciculus, inferior-fronto-occipital fasciculus, superior longitudinal fasciculus, temporal superior longitudinal fasciculus, parietal superior longitudinal fasciculus, superior cortico-striate tract and inferior frontal superior frontal cortex (Cohen's d range: −0⋅016 to −0⋅036, p_FDR_ range: 0⋅049 to 3⋅74 × 10^−4^). Increased DS were associated with reductions of white matter microstructural integrity in the inferior longitudinal fasciculus, superior longitudinal fasciculus, parietal superior longitudinal fasciculus, superior cortico-striate tract (β range: −0⋅021 to −0⋅026, p_FDR_ range: 0⋅045 to 0⋅021).

*Child report:* The only significant association between reports by children and individual brain regions was for surface area of transverse temporal and MDD diagnosis (Cohen's *d* = 0⋅027, p_FDR_ = 0⋅019). No significant association was found for white matter microstructural measures (p_FDR_ > 0⋅116).

### 3.4 The association between adolescent MDD, DS and brain measures controlling for mood of caregivers

We conducted an additional sensitivity analysis to test if associations between caregiver reports of MDD/DS in the children remained significant after controlling for measures of current depression in the caregivers. All associations remained significant for global brain measures (Fig. S5). Results for single brain regions can be found in Figs. S6–7. Overall results with and without controlling for ratings on depressive scale in caregivers showed high correlation (across all association tests between individual brain measures and MDD/depression symptoms reported by children and caregivers, *r* = 0.996 for standardised effect sizes, *r* = 0.984 for *p*-values, see Fig. S8). For those associations that were significant without controlling for mood of caregivers, all remained in the same direction and 90.3% remained significant after FDR-correction.

Additional sensitivity analyses indicated results remained significant after controlling for medication (in child) and were consistent across sites/scanner (see Supplementary Information, Figs. S9–16). Findings also remained robust (in terms of comparison to the other caregiver report results) after controlling for the magnitude of reporter discrepancy (see Figs. S17–19).

### 3.5 Discrepancy between caregiver and child report of depressive symptoms and associations with socio-environmental factors

A significant but low agreement of DS was observed between child and caregiver reports of depression in the child (unweighted Cohen's Kappa = 0.06, *p* = 5⋅29 × 10^−12^). See Fig. S4. Among the caregiver-child pairs, 92 pairs showed large discrepancy (one reported severe DS and the other none), 705 showed moderate discrepancy (discrepancy = two levels) and 7802 pairs showed low or no discrepancy (discrepancy <= one level, contains 7408 pairs that both reported DS lower than mild). Additional analyses that examined reporter discrepancy across different KSADS items indicated a heterogenous pattern of reporter discrepancy across the entire range of diagnostic items. See Fig. S20.

**Fig. 4:**
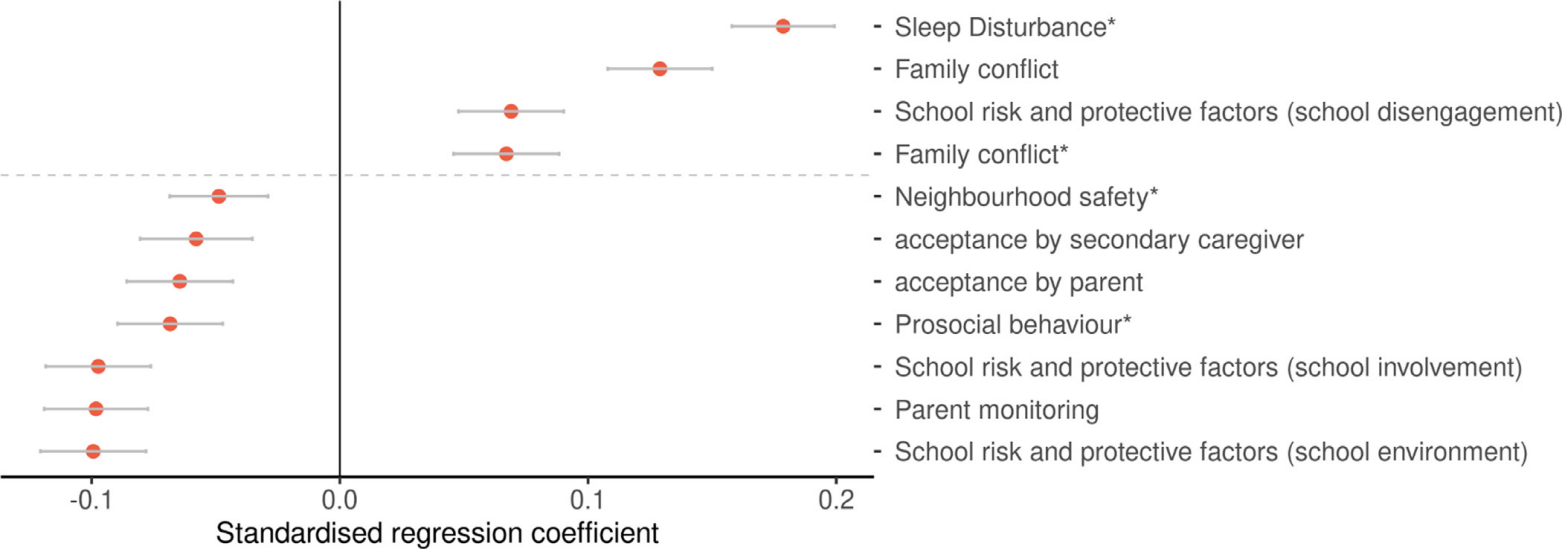
Associations between socio-environmental factors and absolute discrepancies of caregiver and child reports on DS. Variables marked with an asterisk are caregiver reports and the rest are child reports. The *x*-axis shows the standardised regression coefficients. The *y*-axis shows the variables significantly associated with absolute discrepancies of caregiver and child reports (p_bonferroni_< 0.05). Error bars represent 95% confidence interval. A positive regression coefficient represents a positive relationship between the given trait and the absolute discrepancy reported by caregivers and children, and a negative regression coefficient represent a relationship between the trait and less discordance between caregiver and child reports.

Greater discrepancy in caregiver and child report of DS was positively associated with sleep disturbance and family conflict reported by caregivers on children, and family conflict and school disengagement reported by children (β range: 0⋅060 to 0⋅169, p_Bonferroni_ range: 2⋅31 × 10^−5^ to 5⋅26 × 10^−18^). Greater agreement, reflected by negative associations, was found with increased neighbourhood safety and prosocial behaviour of children reported by caregivers, as well as acceptance by caregiver/parent and secondary caregiver, school environment, school involvement and caregiver/parent monitoring reported by children (β range: −0⋅048 to −0⋅096, p_Bonferroni_ range: 3⋅86 × 10^−7^ to 9⋅15 × 10^−56^). See Fig. 4.

## 4 Discussion

The current study leverages the largest available sample to report brain structural differences and their association with caregiver and child reports of depression in early adolescence. We demonstrated that MDD and DS (as reported by caregivers) were associated with similar imaging findings as seen in adult samples, including reduced global cortical volume and global fractional anisotropy (FA) (MDD: Cohen's d range: −0⋅022 to −0⋅027, DS: β range: −0⋅029 to −0⋅057). Our findings also suggest that surface area differences, which have been less consistently reported in adult studies, may be a feature of depressive symptoms in adolescents. Reports of depression in children given by caregivers consistently demonstrated stronger associations with cortical structure and white matter microstructure compared to child report. Finally, reporter discrepancy was positively associated with family conflict and school disengagement (β range: 0⋅060 to 0⋅169). Higher levels of prosocial behaviour in both school and family environments were linked to lower reporter discordance (β range: −0⋅048 to −0⋅096). Sensitivity analyses demonstrated that our results were not related to potential confounders such as anti-depressant medication use (child and caregiver) and scanner and site differences.

Along with the global cortical and white matter differences described above, we also report regionally reduced white matter microstructural integrity in fronto-limbic circuits such as the superior longitudinal fasciculus and cortico-striate tract. This is consistent with previous large scale, population-based studies in adult MDD suggesting that aberrant patterns of white-matter microstructure are present at the early stages of the disease [5]. Reduced microstructural integrity in these association fibres has been previously found to be related to compromised cognitive control, which may underpin clinical features of depression [5,42,43].

The present investigation also found reduced cortical surface area in adolescents with depression, globally and regionally, including a diffuse pattern of localised surface area deficits (MDD: Cohen's *d* = −0⋅021, DS: β = −0⋅066), but not in cortical thickness. Notably, unlike the overall pattern of results, surface area reductions are less commonly reported in adult depression compared to cortical thickness reductions [8]. This therefore implies that surface area reductions may be specifically related to onset and risk factors of depression in early life stages. Similar findings were also shown in another cohort study looking at older adolescents by Schmaal et al. [8]. While not as commonly reported in adult populations as cortical thickness, surface area reductions have shown to be genetically correlated with MDD [44] and are associated with early risk factors of MDD, such as early life trauma and low birth weight [45,46]. Regions that demonstrate cortical surface area abnormalities, such as the precentral gyrus, inferior parietal gyrus, and the superior frontal gyrus may be more vulnerable to the effects of delayed maturation in adolescent depression due to reduced synaptic pruning and dendritic growth over this period [47,48]. Longitudinal studies are needed to understand the origin and development of depression-related, surface area brain features during adolescence and across the life course.

Similarities were observed in the association between depression and brain measures across caregiver and child reports. For example, DS from both reports was significantly associated with global cortical volume (caregiver: β = −0⋅037; child: β = −0⋅027) and surface area (caregiver: β = −0⋅034; child: β = −0⋅020), while MDD status was associated with decreased whole brain FA (caregiver: β = −0⋅030; child: Cohen's *d* = −0⋅021). The effect sizes were similar to those found in adults [6]. However, the current study also revealed that reports of depression by caregivers on adolescents demonstrated stronger and more numerous associations with brain structural measures than by adolescent self-report. These were not biased by medication or by current mood of the caregivers themselves. A difficulty in the diagnosis of depression in adolescents is the integration of reports from both caregivers and adolescents [25,26]. Although we found agreement between caregiver and child reports across individual depressive items, there were indications of important differences. In line with previous work [25,49,50], internalising and somatic type symptoms (e.g., self-esteem, guilt) were more commonly reported by child than caregiver, while decreased concentration and functional impairments were reported more by caregiver than child. Given the stronger neuroimaging associations found for caregiver report of depression, we consider that cognitive and functional impairments may be more strongly connected with these early neurobiological changes — these hypotheses should be tested in future work.

Depressive symptoms reported by caregivers and children showed significant but low correlation, and caregiver's report showed greater associations with brain structural measures. Our findings demonstrate that divergences in origins of reporting relate to environmental and societal factors such as family conflict and social cohesion [51]. These findings reveal the importance of a supportive environment in defining caregiver-child reporter differences; factors such as child-perceived parental support and acceptance also imply secure attachment styles [52]. It is possible that contextual associations between environmental factors and reporter discrepancy may be associated with developmental processes specific to adolescents. Therefore, whilst we cannot completely exclude possible contributions of broader socio-environmental factors, we consider it unlikely that current socio-economic status was driving our main neuroimaging findings, as we have controlled for these in our main analysis. Future longitudinal work should examine the neurobiological consequences of these external societal factors to better understand their role in the origins of the disorder, as well as the potential for environmental intervention.

Although this study benefits from the large imaging sample size, there are limitations. The ABCD cohort is currently a cross-sectional sample. Longitudinal research is needed to facilitate investigating causal effects in these relationships and to inform case-control differences in developmental courses. Further, MDD diagnosis was unavailable in the current data release (2.0.1) for ∼20% of participants due to the inclusion of subclinical participants that did not reach criteria for case or control categorisation (as conducted by ABCD study team). Additional analysis however suggests minimal bias between individuals with and without this missing data (see Table S1 in the Supplementary Information for further detail). Missing data will remain a challenge for community-based population cohorts like ABCD and its treatment will warrant important consideration going forward. While the current study uses both a binary and continuous measure of depression, depressive symptoms are notably highly heterogeneous [53]. This heterogeneity can have pronounced research and clinical implications; for example, individual symptoms may differentially impact impairment of psychosocial functioning [53], and distinctive patterns for longitudinal trajectory of individual symptoms may have heterogeneous underlying neurobiological mechanisms. Future work should examine depressive symptom heterogeneity in the context of brain structure especially during adolescence when subclinical symptoms may manifest, and uncertainties exist around subsequent formal diagnoses.

Although we appreciate the above determinants of heterogeneity for depression, it is important to focus on the neurobiological associations directly linked with the overall diagnosis and severity as a first step, given that disease prediction using neuroimaging phenotypes are predominantly trained and investigated in adult samples [54], [55], [56]. The present findings on the early origins of depression showed distinctive patterns compared to results from adults, which provides strong rationale for separating investigations on diagnosis for adolescence depression, as well as its prediction and treatment. Further, the current findings were generally robust against influence from comorbidity. However, some associations, for example, those found in general cortical grey matter measures, attenuated after controlling for comorbidity. Reasons for this may include shared genetic and environmental risk factors between major psychiatric disorders [57]. Future studies using genetic and epigenetic data may be able to interrogate cross-disorder associations more directly.

Small effect sizes found for the associations in the present study are likely to be contributed by the heterogeneity of disease manifestations and presentation of subtypes. Small effect sizes are a challenge in large neuroimaging research due to the small amount of variance explained by each individual variable [58,59]. However, big data research also allows for the identification of subtle effects, and neurobiological associations with depression are indeed consistently small in large-sample studies [5], [6], [7], [8], [9]. These subtle effects may not be statistically detectable in small-scale studies, which also have the caveat of potentially inflated effect sizes due to sample selection bias [59]. However, the advent of machine learning techniques that examine multiple neuroimaging variables simultaneously in large multi-site studies holds promise of a move towards the identification of clinically relevant neuroimaging disease markers [60]. A further limitation is the young age of the adolescents in the current sample (aged 9–11). It is likely that the cascade of neurobiological changes associated with the onset of puberty may have a further significant impact on the neural circuits implicated in depression [61,62]. Future research is needed to explore any interaction effects between pubertal development, brain measures, and depression in adolescents.

Our findings demonstrate similarities between adult and adolescent imaging features of depression which collectively suggest that cortical and white matter microstructural abnormalities are present early in the disease course of depression and that some of these may extend throughout the lifespan [8]. We demonstrate that these depression-related imaging features are not related to medication in this early adolescent sample. Our results also show evidence of decreased surface area, which may imply adolescent-specific vulnerability. Investigating the origins of these differences may further the understanding of the aetiology of depression over this highly sensitive neurodevelopmental period and thus, help identify at-risk youth. Future longitudinal studies may further inform causal relationships between depression during adolescence and brain structural development.

## Contributors

XS, NM, LR and HCW were responsible for the project conceptualisation, methodology and validation. XS and MJA carried out the data curation. XS, NM and HCW were responsible for the decision to submit. XS was responsible for formal analysis. XS and NM were responsible for writing the original draft and visualisation. XS, NM, SYC, MCB, SML, LR, HCW reviewed versions of the manuscript. XS, NM, LR and HCW were responsible for manuscript editing and review. AMM, SML and HCW were responsible for supervision, project administration, resources, and funding acquisition. XS, NM, MCB, MJA, LR, AMM and HCW had full access to the raw data used in this study.

## Data sharing statement

Data used in the preparation of this article were obtained from the Adolescent Brain Cognitive Development (ABCD) Study® (https://abcdstudy.org), held in the NIMH Data Archive (NDA). A full list of supporters is available at https://abcdstudy.org/federal-partners.html.

Qualified researchers can request access to ABCD shared data through the NDA portal. Scripts for the analyses in this project can be found on this Github repository: https://github.com/xshen796/ABCD_MDD_brain.

## Declaration of Competing Interest

AMM receives two separate Wellcome Trust awards (104036/Z/14/Z and 220857/Z/20/Z), a Sackler trust grant, illumina and Janssen speaker fees. HCW is a co-recipient of a Wellcome Trust award (104036/Z/14/Z). NM is the recipient of a Mental Health Research UK PhD studentship. There are no conflicts of interest declared by other authors.

## Appendix Supplementary materials

Image, application 1

Image, application 2

Image, application 3

Image, application 4

Image, application 5

Image, application 6

Image, application 7

Image, application 8
