# Supplementary material for: Brain structural associations with depression in a large early adolescent sample (the ABCD study®)
Source: eClinicalMedicine. 2021 Nov 20;42:101204. doi: 10.1016/j.eclinm.2021.101204 (PMC8608869; doi:10.1016/j.eclinm.2021.101204)
Supplement: Supplementary file 1 [file mmc1.docx]

**Supplementary Information**

**Brain structural associations with depression in a large early adolescent sample (the ABCD cohort)**

Shen *et al.*

**Supplementary methods and results**

**Scanning protocols**

Protocols used for data acquisition and processing were described elsewhere^1,2^. In brief, T1-weighted data was acquired by magnetisation-prepared rapid acquisition gradient echo scans with a resolution of 1×1×1 mm^3^, which was used for generating cortical structural measures, and diffusion-weighted data was obtained by high angular resolution diffusion imaging scans, used for generating white matter microstructural measures.

**Unrelated participants**

Unrelated participants were selected by keeping the first entry of each individual family ID (rel_group_id: <https://nda.nih.gov/general-query.html?q=query=data-element%20~and~%20searchTerm=name:%20%22rel_group_id%22>).

**QC criteria for brain structural measures**

For cortical measures, QC measures on raw imaging was first applied to remove poor-quality raw T1 scans (data field: ‘iqc_t1_ok_ser’). Participants that had low post-processing quality check scores (<1) for Freesurfer outcome identified (data field: ‘fsqc_qc’) were then removed.

For white matter microstructural measures, participants with poor-quality raw diffusion imaging scans (data field: ‘iqc_dmri_ok_ser’), poor-quality raw T1 scans (data field: ‘iqc_t1_ok_ser’) and poor quality of FreeSurfer parcellation for T1 data (data field: ‘fsqc_qc’, potentially indicating low quality for T1 scans) were removed from further analyses. We also removed subjects that had low score for post-processing (data field: ‘dmri_dti_postqc_qc’). Finally, as there were extreme values that caused the distributions of global white matter measures heavily skewed, we removed participants showing global FA and MD values 5 standard deviations from mean (see Figure S2 for sample sizes and Figure S3 for distribution before and after removing outliers).

We used the data field ‘dmri_dti_postqc_qc’ for screening data after preprocessing. The variable is a binary variable, covering 3733 people. Among them, 224 did not pass QC and 3509 passed. To test the impact of post-processing QC, first we showed the standardised values of global fractional anisotropy (FA) and mean diffusivity (MD) (after removing outlying values of -/+ 5 std away from mean) in Figure S3. Secondly, we conducted analysis of this subsample (N=3733) with post-processing QC data available and for those who passed QC in this subsample (N=3509). The main model of testing associations between caregiver/child report of MDD/depressive symptoms (DS) and regional brain measures was used. As shown in Figure S21, overall correlation of the standard regression coefficient/Cohen’s d for all the tests conducted were highly correlated (r=0.918). The uncorrected p-values were also highly correlated (r=0.789).

**Deriving life-time MDD definition from current and past MDD status**

The curated data from ABCD (Adolescent Brain Cognitive Development) study contains three MDD (Major Depressive Disorder) definitions: MDD current (field names: ksads_1_840_t and ksads_1_840_p for reports by children and caregivers, respectively), MDD past (field names: ksads_1_842_t and ksads_1_842_p for reports by youths and caregivers respectively) and MDD current in partial remission (field names: ksads_1_841_t and ksads_1_841_p). When defining the lifetime MDD definitions, participants who were ***either*** a case for MDD current or past definition would be identified as cases, and those who were controls for ***both*** current and past MDD definitions were identified as controls. Those who were cases for MDD current in partial remission were also identified as cases. Life-time definitions were derived for reports by caregivers and youths separately.

**Deriving total scores of DS**

DS were generated based on DSM-V (Diagnostic and Statistical Manual of Mental Disorders, 5^th^ edition)^3^ criteria for the severity scale of. For each item, a binary outcome indicates whether the single symptom met clinical significance (1=Yes and 0=No). For each item, a life-time score was generated using the same method for generating lifetime MDD definition. The life-time score was then used for generating DS.

In total there were 28 items used, which covered 15 individual DS (see Table S2). Among the 15 individual DS, depressed mood, anhedonia and fatigue were core symptoms and the rest of 12 DS were secondary symptoms. Life-time scores of these individual DS were used to generate a total measure of DS that includes four severity levels: severe, moderate, mild and none of the above. A detailed description of the total measure of DS can be found in Table S3.

**Agreement between caregiver and child report**

We tested the agreement between caregiver and child report. Proportion of agreement for MDD diagnosis between caregiver and child report was estimated using the ‘agree’ function in R package ‘irr’ (version 0.84.1, <https://cran.r-project.org/web/packages/irr/irr.pdf>). Tolerance of disagreement was set as 0. Cohen’s Kappa of DS between caregiver and child report was conducted using the ‘kappa2’ function from the ‘irr’ R package (version 3.6.2). For both analyses, only participants with non-empty values for both caregiver and child reports were included (N_MDD definition_=8635, N_DS measure_=8599).

Proportion of agreement between caregiver and child report of MDD diagnosis was 95.9% (Table 2). Among all participants, 8273 were identified as controls by both child and caregiver report (95.8% of the total sample), 182 were cases based on caregiver report but not child report (2.11%), 168 were cases according to child report but not caregiver (1.95%), and finally 12 were cases according to both reports (0.14%). Agreement between caregiver and child reported DS was reported in the main text.

**Average and discrepancy of DS reported by caregivers and children and its association with brain structural measures**

Average reports for the severity of depression was generated for each child-caregiver pair. Results for the associations between the average severity and general/regional brain measures are shown in Figures S17-19.

Discrepancy was generated by obtaining the absolute values of subtracting caregiver and child reports of DS (Figure S4). Associations between discrepancy of DS and general brain structural measures were tested (Figure S22). For regional measures, the associations with discrepancy of DS were tested on those brain measures that associated with caregiver report of depression (Figure S23).

**Validating DS measured by KSADS and CBCL**

In the present cohort, two types of mental health scales were used. Although we reported results based on KSADS, we tested DS assessed by CBCL (The Child Behaviour Checklist) as an additional analysis and compared the results produced using KSADS and CBCL. DS assessed using CBCL was based the DSM-5-oriented items.

KSADS-derived scores of DS reported by caregivers showed high correlations with CBCL’s depression score based on the DSM-5 (Diagnostic and Statistical Manual of Mental Disorders, 5th Edition) scale (r=0.346). The KSADS DS reported by youths showed weak correlation with CBCL depression score (r=0.076). See Figures S24.

CBCL DMS-5-oriented score of depression reported by caregivers showed consistent results with DS assessed by KSADS reported by caregivers (Figures S25-26). Associations were found in general cortical surface area, volume and white matter FA (β ranged from -0.025 to -0.043, p ranged from 1.86×10^-3^ to 1.53×10^-6^, Figure S25). Overall effect sizes for regional brain measures showed high correlation with results for KSADS-measured DS (r = 0.713, see Figure S26).

**Depressive problems of caregivers**

A subscale of DSM-5-oriented items for depressive problems from the Adult Self-Report (ASR) in the Achenbach System of Empirically Based Assessment was used for the one caregiver who accompanied the child to the study. We used the field ‘asr_scr_depress_r’ as a score for severity of depression in caregivers (N=8633). A total of 14 items were used for this scale^4^. There were five participants who did not answer any of the questions, and thus they were removed from analysis. All other participants completed the entire questionnaire. This scale of DS in caregivers was tested to indicate recent DS, and a high proportion of reporters were mothers (85.66% of all caregiver reporters, N_Mother_=7412).

We tested the agreement between the ASR scale and self-reported history of depression using glm model, setting the binary variable of self-reported history as independent variable, and the ASR scale as dependent variable. Two measures for maternal risk showed the greatest agreement (OR=0.762, p<2×10^-16^), followed by measures for caregivers (OR=0.556, p<2×10^-16^). Measures for paternal risk showed the poorest agreement (OR=0.536, p<4.49×10^-7^).

**Recent social deprivation**

A measure of recent social deprivation was included in the analysis as a covariate. The measure was derived from items the parent demographics survey (<https://nda.nih.gov/data_structure.html?short_name=abcd_lpds01>), which was answered by parents. Participants were asked to give a ‘Yes’ (=1) or ‘No’ (=0) answer to seven questions: 1. Needed food but couldn't afford to buy it or couldn't afford to go out to get it? 2. Were without telephone service because you could not afford it? 3. Didn't pay the full amount of the rent or mortgage because you could not afford it? 4. Were evicted from your home for not paying the rent or mortgage? 5. Had services turned off by the gas or electric company, or the oil company wouldn't deliver oil because payments were not made? 6. Had someone who needed to see a doctor or go to the hospital but didn't go because you could not afford it? And 7. Had someone who needed a dentist but couldn't go because you could not afford it?

The sum score of all the ‘Yes’ answers was used as the measure for social deprivation. The measure has a mean value of 0.936, with its minimum and maximum scores = 0 and 14, respectively. We included this variable as the main proxy that may confound MDD-related deficits in brain development instead of controlling more extensively for socio-environmental protective/risk factors, as the latter would have restricted our sample with complete data to less than half of the current sample (N=4,036).

**Sensitivity analyses: site differences**

Data was collected in 22 sites across the United States. In order to test whether site difference makes significant impact on data, we used leave-one-out method to test the robustness of results. This was conducted on associations between MDD/DS and general brain measures.

For each association (e.g. cortical volume ~ MDD reported by caregivers), the analysis was conducted 22 times. Each time, data from one site was taken out, and therefore analysis was performed on the remaining 21 sites. Effect sizes of leave-one-out analysis were then compared against the one found using the entire sample. Results are reported in Figures S9-12.

Impact of differences in scanning site were assessed using leave-one-out analysis for the significant associations found between MDD diagnosis/DS and general brain measures in the total sample, described above were assessed (Figures S9-12).

*Caregiver report:* All associations between MDD diagnosis/DS reported by caregivers and brain measures remained significant in all iterations of the leave-one-out analysis (p ranged from 0.037 to 3.44×10^-9^). Therefore, the results regarding caregiver report are not likely to be driven by a single site.

*Child report:* Associations between DS reported by child and brain measures were also significant in all iterations (p ranged from 0.034 to 1.72×10^-4^). For associations between MDD diagnosis reported by child and brain measures, 14 out of 22 iterations were significant (p ranged from 0.049 to 0.021) and 8 iterations were not significant (p ranged from 0.091 to 0.053) for sulcal depth, and 19 iterations were significant (p ranged from 0.047 to 0.007) and 3 were not significant (p ranged from 0.082 to 0.069) for FA. In summary, results for DS reported by child are not likely to be driven by sites, whereas results for MDD diagnosis are more heterogeneous across sites.

**Sensitivity analysis: scanner differences**

As the three type of scanners from the major manufacturers can show significant impacts on the estimation of intracranial volume^5^, we added scanner manufacturer as an additional covariate in a secondary sensitivity model. All significant associations found between general brain measures and MDD/DS remained significant (see Figure S13). Effect sizes for the associations between individual brain structural measures and MDD/DS showed high correlation with results of the main model (r = 0.999 for standardised effect sizes, r = 0.996 for p-values, see Figure S14).

**Sensitivity analysis: impact of medication**

The use of antidepressants was small in this sample (N=136). However, we also investigated the potential impact of these on our main finding by including the use of antidepressants as an additional covariate in a sensitivity analysis.

The ABCD Parent Medications Survey Inventory Modified from PhenX (short name: medsy01, URL: <https://nda.nih.gov/data_structure.html?short_name=medsy01>) was used to derive a variable for medication usage. The survey was used for caregivers to report medication intake by child in the past two weeks before the assessment took place. In order to estimate the effect of medication on brain imaging measures, we extracted reported medication intake that match the assessment date with the imaging assessment.

We used two types of data columns for screening medication usage. The first one is the general screening question, ‘Did your child take any medications in the past two weeks and if so did you bring them with you?’ (field name: brought_medications, URL: <https://nda.nih.gov/general-query.html?q=query=data-element%20~and~%20searchTerm=name:%20%22brought_medications%22>). Available answers were: 0 = Yes (medications taken in the past two weeks); 1 = Yes (medication brought with the caregiver); 2 = Refused; and 3 = Took No Medications. The second type of screening questions were reported medication names. Data columns with names of prescribed medications were used (field names: med1_rxnorm_p, med2_rxnorm_p, …, med15_rxnorm_p). A list of drug names were generated using the British National Formulary-70 (BNF 70) (<https://www.bnf.org/products/books/>) under the category of depression^6^. We used these drug names as key words to find matched medications as antidepressants.

Combining the two types of questions, we generated a categorical variable to indicate medication usage:

**0** = no medication brought (brought_medications==3) & no prescribed medication name reported (N=5320),
**1** = medication was used for the past two weeks & no prescribed medication name reported (N=2665),
**2** = medication was used for the past two weeks & at least one medication reported & none was included in the list of antidepressants (N=1942), and finally,
**3** = medication was used for the past two weeks & at least one antidepressant was reported (N=136).

In the sensitivity analysis, this variable of medication usage was included as an additional covariate. Results for the association between general brain measures and MDD/DS can be found in Figure S15. Comparison of effect sizes and p values between the main model and the model controlling for medication usage, associated with individual brain measures can be found in Figure S16.

All associations found between general brain measures and MDD/DS remained significant (Figure S15). The effect sizes and p-values for the associations between measures of individual brain structures and MDD/DS were highly correlated with the results of the main model (r=0.989 for standardised effect sizes, r=0.965 for p-values, see Figure S16).

**Sensitivity analysis: covarying comorbidity**

We further looked at comorbid major psychiatric conditions (Bipolar I, Bipolar II, ADHD, Psychosis and Conduct disorder) reported by caregivers. Within the 194 cases, 132 reported one or more of these conditions in the present/past (68.04%) and 62 reported none (31.96%). There were 1612 MDD controls reported any of the above conditions (24.12%) and 5072 reported none of these conditions, including MDD (75.88%).

Further, a supplementary analysis was conducted to see if covarying for comorbidity may change the main findings regarding the associations between brain structural measures and parent report of depression. The additional covariate comorbidity was generated by summarising the comorbid major psychiatric conditions (1=with any comorbid condition and 0=none of the comorbid conditions reported).

Results can be found in Figures S27-28.

**Measures for socio-environmental factors**

Measures from ABCD sum scores culture & environment – caregiver (abcd_sscep01), ABCD sum scores culture & environment – youth (abcd_sscey01) and ABCD sum scores physical health – caregiver (abcd_ssphp01) were used. The scores for culture and environment reported by both caregivers and children were used because the questionnaires contain different items.

Item screening was conducted by removing items that have less than half of the total sample sizes (Discrimination Measure: dim_y_ss_mean and Mind Diet score: cna_p_ss_sum were thus removed from analysis). As we used the entire sample, therefore questionnaires that focus on one specific culture were not included in the analysis (ABCD Parent Mexican American Cultural Values Scale). For each participant, those that answered less than half of the questionnaire items in a single scale were set as NA for the given summary score.

A complete list of variables included in the analysis can be found in Table S4.

**References**

1 Lopez-Leon S, Janssens AC, Gonzalez-Zuloeta Ladd AM, *et al.* Meta-analyses of genetic studies on major depressive disorder. *Mol Psychiatry* 2008; **13**: 772–85.

2 Garavan H, Bartsch H, Conway K, *et al.* Recruiting the ABCD sample: Design considerations and procedures. *Dev Cogn Neurosci* 2018; **32**: 16–22.

3 American Psychiatric Association. Diagnostic and statistical manual of mental disorders (5th edition). 2013.

4 Barch DM, Albaugh MD, Avenevoli S, *et al.* Demographic, physical and mental health assessments in the adolescent brain and cognitive development study: Rationale and description. Dev. Cogn. Neurosci. 2018; **32**: 55–66.

5 Casey BJ, Cannonier T, Conley MI, *et al.* The Adolescent Brain Cognitive Development (ABCD) study: Imaging acquisition across 21 sites. Dev. Cogn. Neurosci. 2018; **32**: 43–54.

6 Shen X, Adams MJ, Ritakari TE, Cox SR, McIntosh AM, Whalley HC. White Matter Microstructure and Its Relation to Longitudinal Measures of Depressive Symptoms in Mid- and Late Life. *Biol Psychiatry* 2019; **86**: 759–68.

Table S1. Sample sizes and demographic features for those with and without MDD.

|  |  |  | **N** | **Age** | | **Sex (% of Male)** |
| --- | --- | --- | --- | --- | --- | --- |
|  |  |  |  | Mean | SD |  |
| **MDD** | **Reported by caregivers** | **Case + control** | 6878 | 9.9 | 0.62 | 51.7% |
|  |  | **Missing data** | 1757 | 9.97 | 0.62 | 54.9% |
|  | **Reported by children** | **Case + control** | 6924 | 9.9 | 0.62 | 51.6% |
|  |  | **Missing data** | 1710 | 9.97 | 0.62 | 55.4% |

Table S2. Items in the scale to assess Major Depressive Disorder (MDD) and depressive symptoms using the Kiddie Schedule for Affective Disorders and Schizophrenia (KSADS). When depressive symptoms were calculated, these variables were counted as one rather than two variables: insomnia and hypersomnia, decreased and increased appetite, weight loss and weight gain, and finally, psychomotor agitation and retardation.

| **Field name** | | | | **DSM-V symptoms** | **Sub-items** |
| --- | --- | --- | --- | --- | --- |
| **Caregiver** | | **Youth** | |  |  |
| **Past** | **Current** | **Past** | **Current** |  |  |
| ksads_1_1_p | ksads_1_2_p | ksads_1_1_t | ksads_1_2_t | Depressed mood (core symptom) | -- |
| ksads_1_5_p | ksads_1_6_p | ksads_1_5_t | ksads_1_6_t | Anhedonia (core symptom) | -- |
| ksads_1_159_p | ksads_1_160_p | ksads_1_159_t | ksads_1_160_t | Fatigue (core symptom) | -- |
| ksads_1_161_p | ksads_1_162_p | ksads_1_161_t | ksads_1_162_t | Concentration disturbance | -- |
| ksads_1_181_p | ksads_1_182_p | ksads_1_181_t | ksads_1_182_t | Decreased self-esteem | -- |
| ksads_1_177_p | ksads_1_178_p | ksads_1_177_t | ksads_1_178_t | Guilt | -- |
| ksads_1_179_p | ksads_1_180_p | ksads_1_179_t | ksads_1_180_t | Hopeless | -- |
| ksads_1_183_p | ksads_1_184_p | ksads_1_183_t | ksads_1_184_t | Impairment in functioning due to depression | -- |
| ksads_1_163_p | ksads_1_164_p | ksads_1_163_t | ksads_1_164_t | Indecision | -- |
| ksads_1_3_p | ksads_1_4_p | ksads_1_3_t | ksads_1_4_t | Irritability | -- |
| ksads_1_157_p | ksads_1_158_p | ksads_1_157_t | ksads_1_158_t | Disturbed sleep | Hypersomnia |
| ksads_22_141_p | ksads_1_156_p | ksads_22_141_t | ksads_1_156_t |  | Insomnia when depressed |
| ksads_1_171_p | ksads_1_172_p | ksads_1_171_t | ksads_1_172_t | Changed weight | Weight gain |
| ksads_1_167_p | ksads_1_168_p | ksads_1_167_t | ksads_1_168_t |  | Weight loss |
| ksads_1_165_p | ksads_1_166_p | ksads_1_165_t | ksads_1_166_t | Changed appetite | Decreased appetite |
| ksads_1_169_p | ksads_1_170_p | ksads_1_169_t | ksads_1_170_t |  | Increased appetite |
| ksads_1_173_p | ksads_1_174_p | ksads_1_173_t | ksads_1_174_t | Psychomotor symptoms | Psychomotor agitation in depressive disorder |
| ksads_1_175_p | ksads_1_176_p | ksads_1_175_t | ksads_1_176_t |  | Psychomotor retardation |
| ksads_23_953_p | ksads_23_964_p | ksads_23_953_t | ksads_23_964_t | Self-harm and suicidal thoughts/attempts | Aborted attempt |
| ksads_23_952_p | ksads_23_963_p | ksads_23_952_t | ksads_23_963_t |  | Interrupted attempt |
| ksads_23_951_p | ksads_23_962_p | ksads_23_951_t | ksads_23_962_t |  | Preparatory actions toward imminent suicidal behavior |
| ksads_23_945_p | ksads_23_956_p | ksads_23_945_t | ksads_23_956_t |  | Self-Injurious behavior without suicidal intent |
| ksads_23_949_p | ksads_23_960_p | ksads_23_949_t | ksads_23_960_t |  | Suicidal ideation active intent |
| ksads_23_948_p | ksads_23_959_p | ksads_23_948_t | ksads_23_959_t |  | Suicidal ideation active method |
| ksads_23_947_p | ksads_23_958_p | ksads_23_947_t | ksads_23_958_t |  | Suicidal ideation active non-specific |
| ksads_23_950_p | ksads_23_961_p | ksads_23_950_t | ksads_23_961_t |  | Suicidal ideation active plan |
| ksads_23_946_p | ksads_23_957_p | ksads_23_946_t | ksads_23_957_t |  | Suicidal ideation passive |
| ksads_23_954_p | ksads_23_965_p | ksads_23_954_t | ksads_23_965_t |  | Suicide attempt |

Table S3. Measure of DS derived from individual items of DSM-V MDD symptomology and diagnosis of suicidality.

| **Severity level** | **Criteria: (condition (a) OR condition (b)) AND condition (c)** | | |
| --- | --- | --- | --- |
|  | **Condition (a)** | **Condition (b)** | **Condition (c)** |
| Severe | Core symptoms = 3 +  Secondary symptoms > 3 | Any suicidal attempt | Identified as case |
| Moderate | Core symptoms >1 +  Secondary symptoms = 2 to 3 | Total symptoms = 7 to 8 | Not identified as Severe |
| Mild | Core symptoms >1 +  Secondary symptoms = 1 to 2 | Total symptoms = 5 to 6 | Not identified as Moderate/Severe |
| None of the above | None of the above | | |

Table S4. Measures of cultural and social environment, family environment, physical health and sociodemographic status. Field names are the column names used in the original ABCD curated data. For those measures that used multiple data fields, notes are provided for the methods of creating them.

| **Field name(s)** | **Description** | **Category** | **Note** |
| --- | --- | --- | --- |
| meim_p_ss_total | Caregiver: Multi-group ethnic identity (total scale) | Cultural and Social Environment |  |
| via_p_ss_hc | Caregiver: Vancouver index of acculturation (heritage culture) | Cultural and Social Environment |  |
| via_p_ss_amer | Caregiver: Vancouver index of acculturation (American 'mainstream' culture) | Cultural and Social Environment |  |
| nsc_p_ss_mean_3_items | Caregiver: Neighbourhood safety | Cultural and Social Environment |  |
| srpf_y_ss_ses | Child: School risk and protective factors (school environment) | Cultural and Social Environment |  |
| srpf_y_ss_iiss | Child: School risk and protective factors (school involvement) | Cultural and Social Environment |  |
| srpf_y_ss_dfs | Child: School risk and protective factors (school disengagement) | Cultural and Social Environment |  |
| fes_p_ss_fc_pr | Caregiver: Family conflict | Family Environment |  |
| psb_p_ss_mean | Caregiver: Prosocial behaviour | Family Environment |  |
| pmq_y_ss_mean | Child: Parent monitoring | Family Environment |  |
| fes_y_ss_fc_pr | Child: Family conflict | Family Environment |  |
| psb_y_ss_mean | Child: Prosocial behaviour | Family Environment |  |
| crpbi_y_ss_parent | Child: acceptance by parent | Family Environment |  |
| crpbi_y_ss_caregiver | Child: acceptance by secondary caregiver | Family Environment |  |
| sds_p_ss_total | Caregiver: Sleep Disturbance Scale (total scale) | Physical Health |  |
| pds_p_ss_female_category, pds_p_ss_male_category | Caregiver: Pubertal development scale | Physical Health | Female and male pubertal development scores were originally separated. |
| demo_comb_income_v2 | Household income | Sociodemographic status |  |
| parent1_edu, parent2_edu | Highest education of parents | Sociodemographic status | A higher education between two caregivers was extracted as the highest education of the household/between caregivers |

Table S5. Statistical models used for association tests between MDD/DS of depression and brain structural measures.

| **Type** | **Measures** | **Number of variables** | **Covariates (MDD/Depressive symptoms)** |
| --- | --- | --- | --- |
| General brain measures | Mean whole-brain cortical thickness | 1 unilateral | age+age2+sex+motion(fsqc_qu_motion)+site+race/ethnicity+recent social deprivation |
|  | Total whole-brain surface area | 1 unilateral |  |
|  | Mean whole-brain sulcal depth | 1 unilateral |  |
|  | Total whole-brain volume | 1 unilateral |  |
|  | Global total white matter fractional anisotropy | 1 unilateral |  |
|  | Global total white matter mean diffusivity | 1 unilateral |  |
| Regional brain measures | Cortical thickness | 34 bilateral | age+age2+sex+motion(fsqc_qu_motion)+site+race/ethnicity+recent social deprivation+intracranial volume |
|  | Cortical surface area | 34 bilateral |  |
|  | Cortical sulcal depth | 34 bilateral |  |
|  | Cortical volume | 34 bilateral |  |
|  | White matter fractional anisotropy | 14 bilateral | age+age2+sex+motion(fsqc_qu_motion)+site+race/ethnicity+recent social deprivation |
|  | White matter mean diffusivity | 14 bilateral |  |

Figure S1. Density plot of global measures of cortical surface area, thickness, volume and sulcal depth. The x-axis represents the standardised scores. The y-axis represents distribution density. For illustration purpose, the plots were generated using the ‘ggplot’ function in R package ‘ggplot2’, with a smoothing adjustment of 2 and an illustration alpha (for transparency) of 0.1.


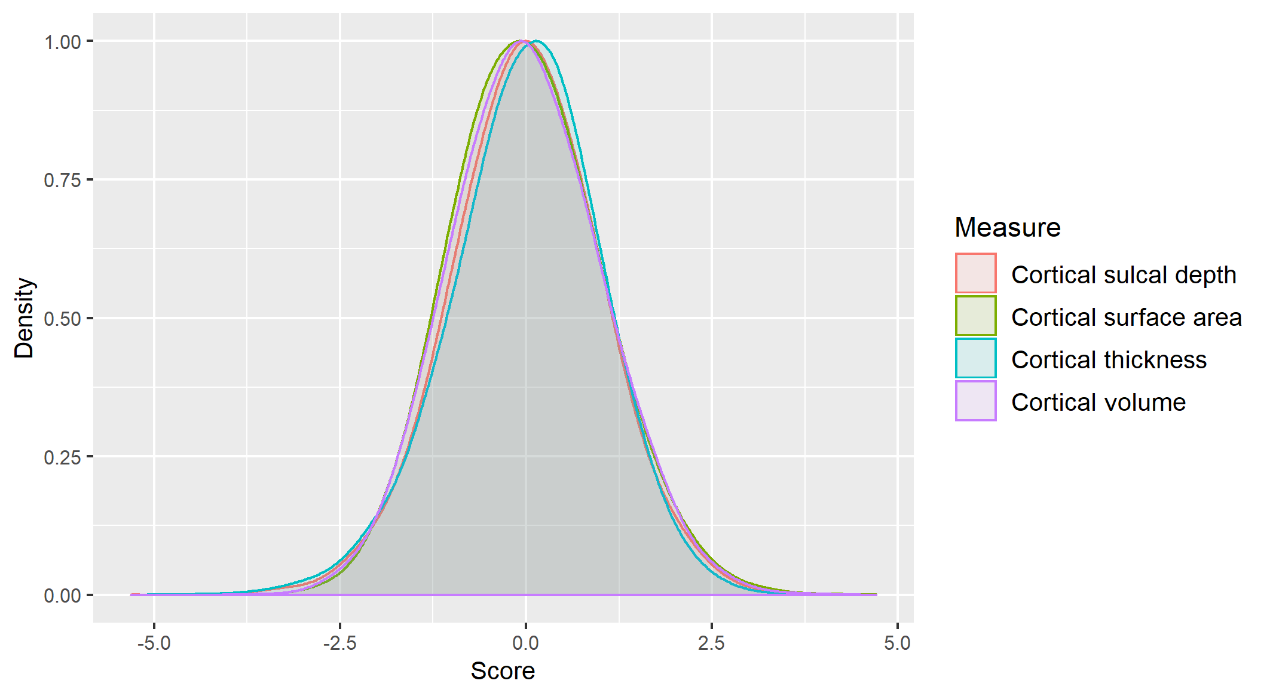


Figure S2. Sample sizes after each quality check (QC) step. For replication purpose, here in this chart we presented the field names used in the ABCD cohort. A general description of the fields used can be found in the Supplementary Methods. Detailed descriptions of each field can be found in the ABCD data dictionary (MRI raw data QC: <https://nda.nih.gov/data_structure.html?short_name=mriqcrp102> and <https://nda.nih.gov/data_structure.html?short_name=mriqcrp202>; Freesurfer QC – cortical measures: <https://nda.nih.gov/data_structure.html?short_name=freesqc01>; dMRI post processing: <https://nda.nih.gov/data_structure.html?short_name=dmriqc01>) and in the quality check documentation (<http://dx.doi.org/10.15154/1503209>).


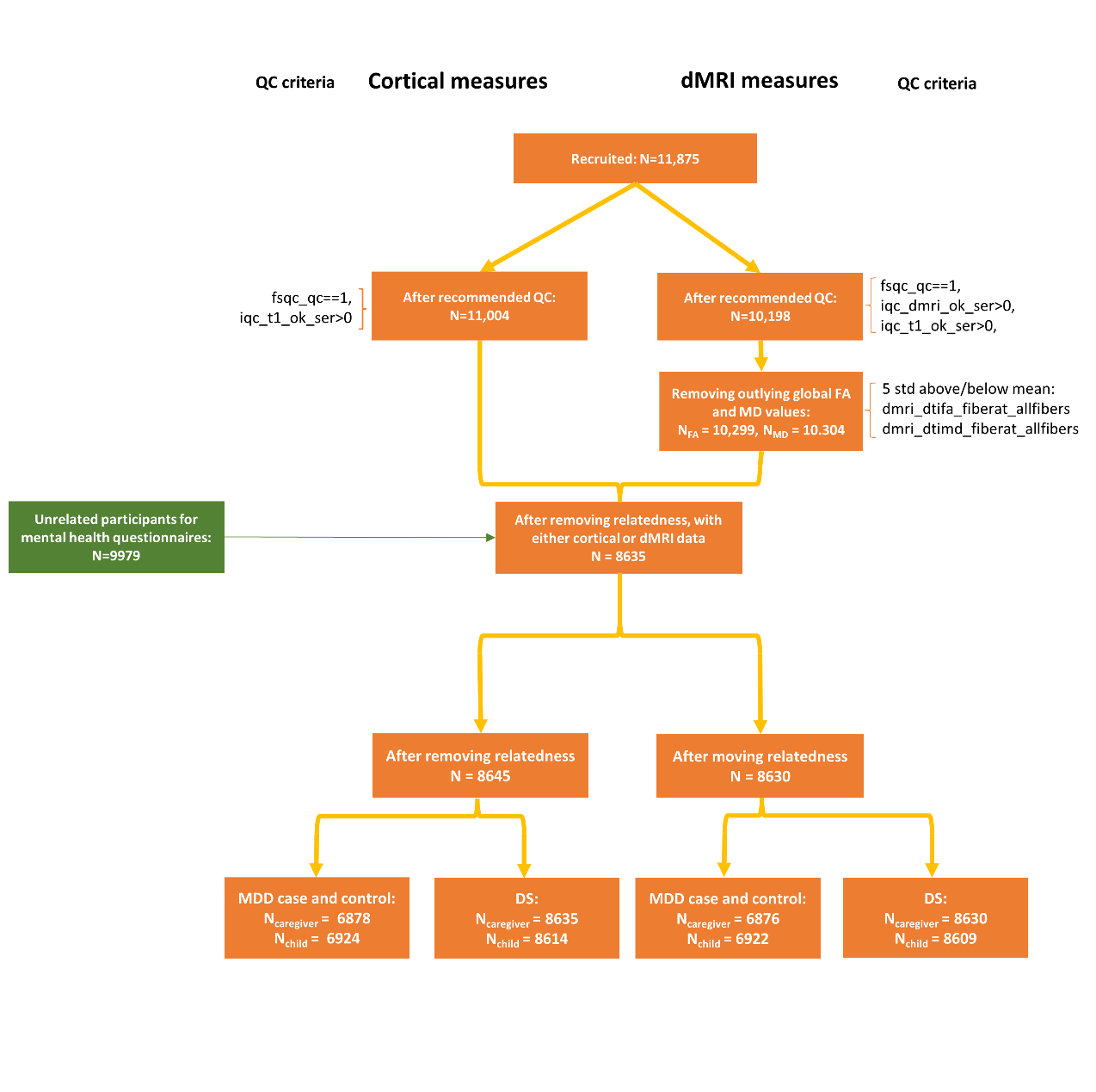


Figure S3. (a) Density plot of global measures of white matter fractional anisotropy (FA) and mean diffusivity (MD). The x-axis represents the standardised scores. The y-axis represents distribution density. In the left panel, data after removing those with poor data quality was used. As there were extreme values causing the distributions heavily skewed, we then removed participants with global values 5 standard deviations away from mean, which results in the distribution maps in the right panel. For illustration purpose, the plots were generated using the ‘ggplot’ function in R package ‘ggplot2’, with a smoothing adjustment of 2 and an illustration alpha (for transparency) of 0.1. (b) Comparison between the standardised values for general white matter microstructural measures that were kept or removed due to post-processing quality check (QC). Red dots represent values outside of 95% confidence interval.


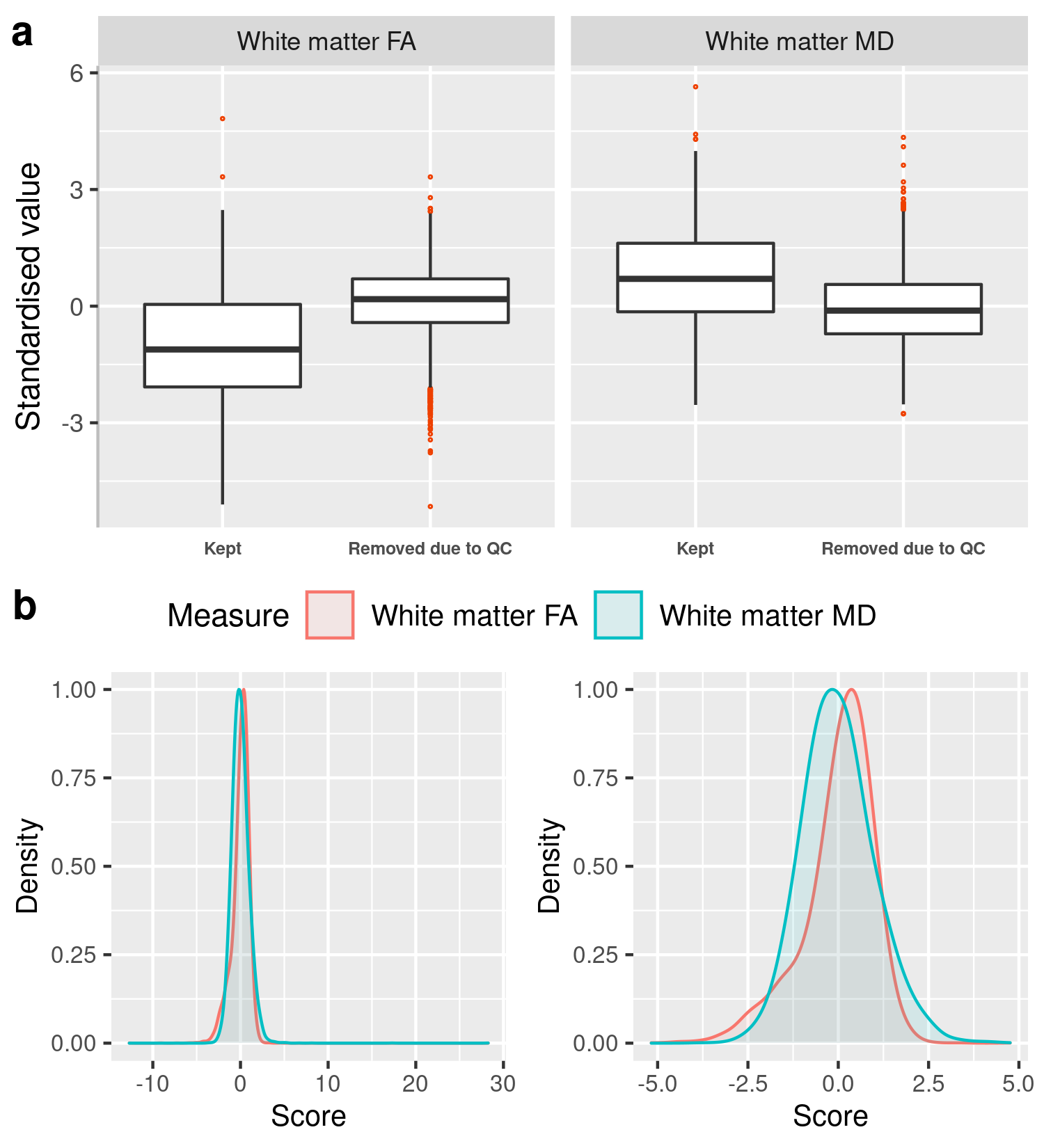


Figure S4. Histogram of DS reported by caregivers and children, absolute discrepancy and discrepancy between caregiver and child reports. The x-axis represents DS in panel a. In panels b and c the x-axes represent the absolute discrepancy and discrepancy between caregiver and child (caregiver - child) reports respectively. The y-axes represent distribution density.


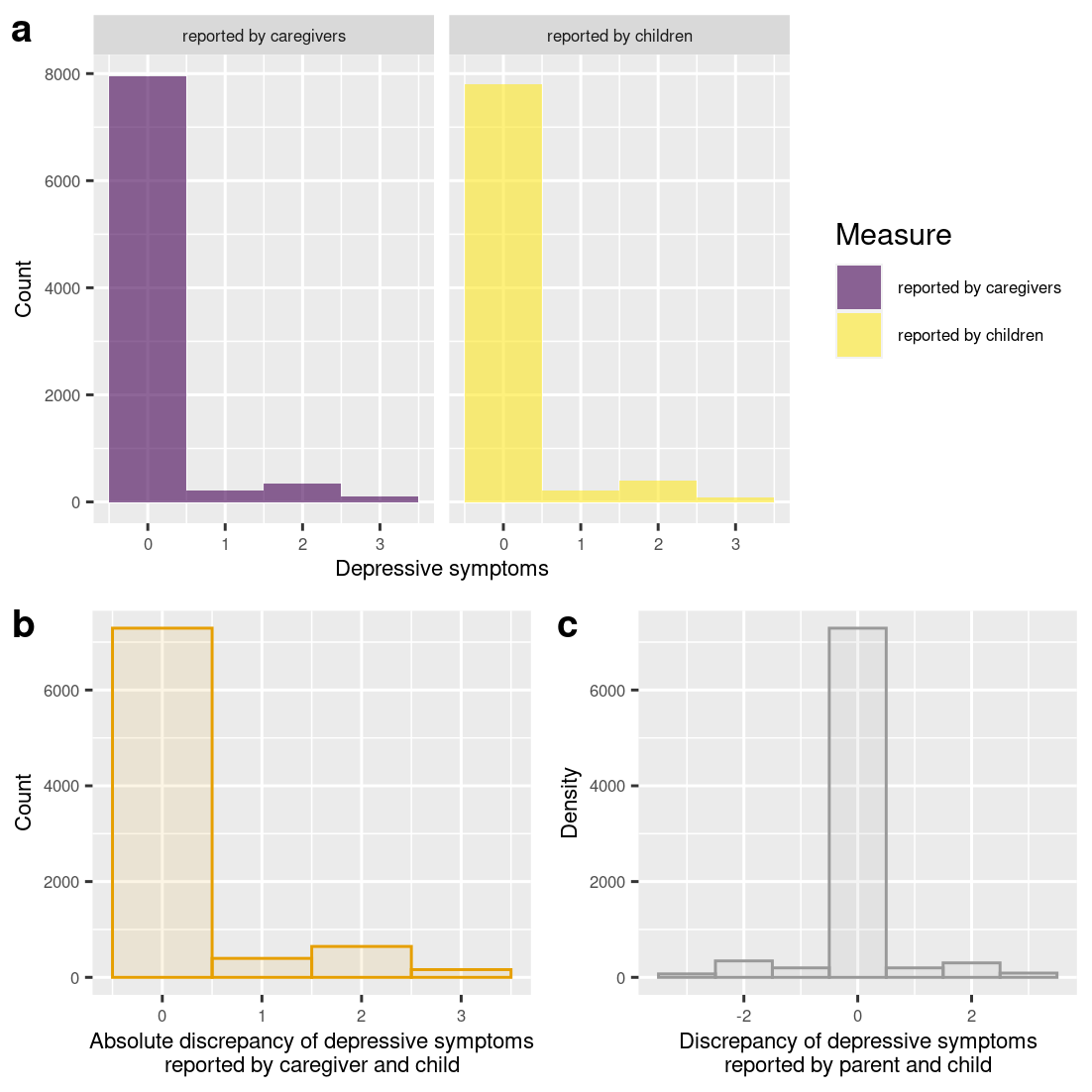


Figure S5. Associations between MDD, depressive symptoms and general measures of brain structures, controlling for ASR scale for severity of depression in caregivers. X-axes represent standardised effect sizes with error bars showing 95% confidence interval, and y-axes represent each general measure of brain structure. Panel a shows the results for MDD/depressive symptoms reported by caregivers on children, and panel b shows the results for symptoms reported by children themselves.


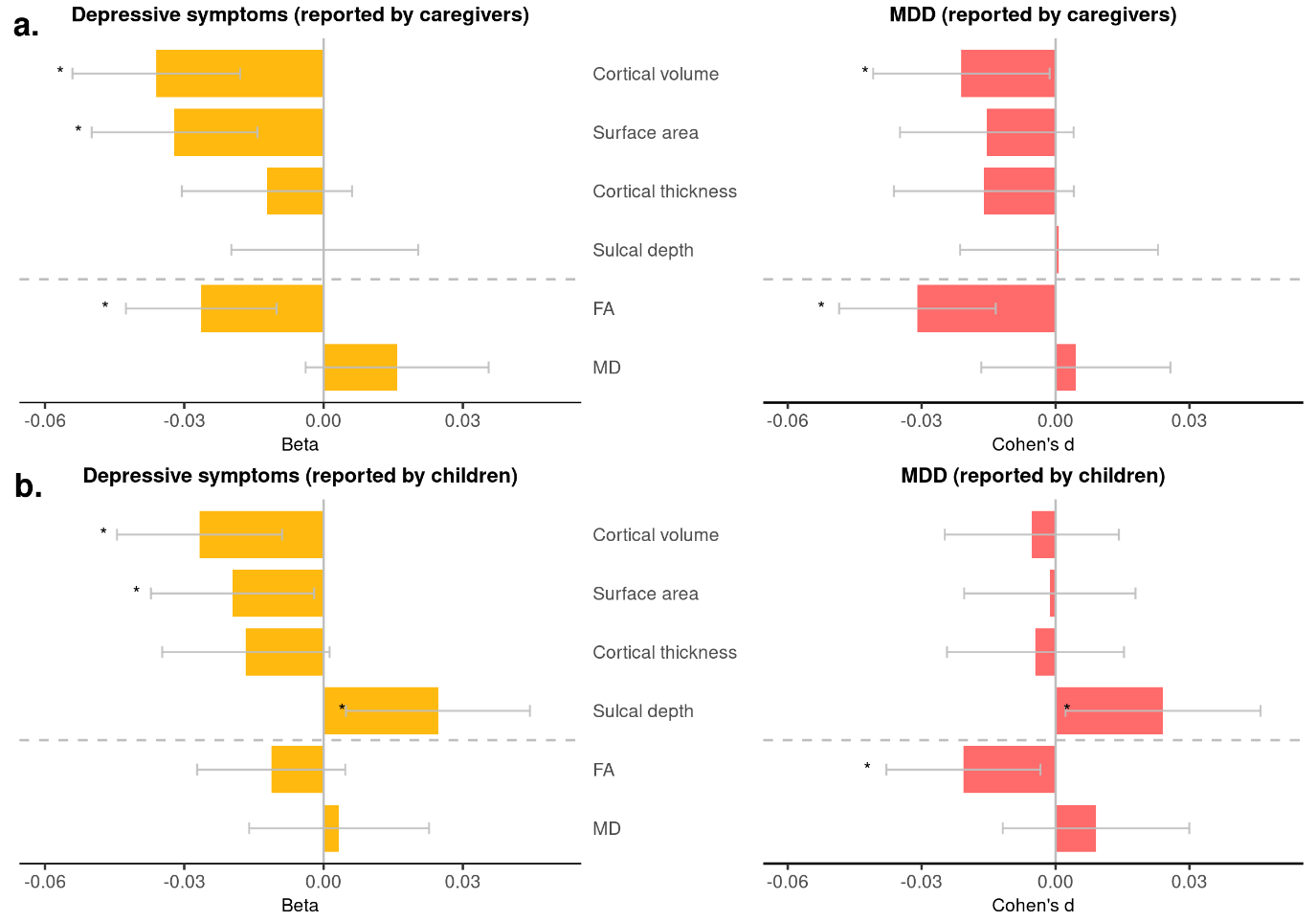


Figure S6. P-value plots for associations between depressive symptoms and measures for single brain regions, controlling for ASR scale for severity of depression in caregivers. X axes represent measures for brain structural measures, and y axes represent -log10 transformed p-values. Panel (a) shows the results for depressive symptoms reported by caregivers on children, and panel (b) shows the results for symptoms reported children themselves. Solid dots represent variables associated with depressive symptoms after FDR-correction. For clarity, threshold for significance after FDR-correction is shown as the pink dashed lines.


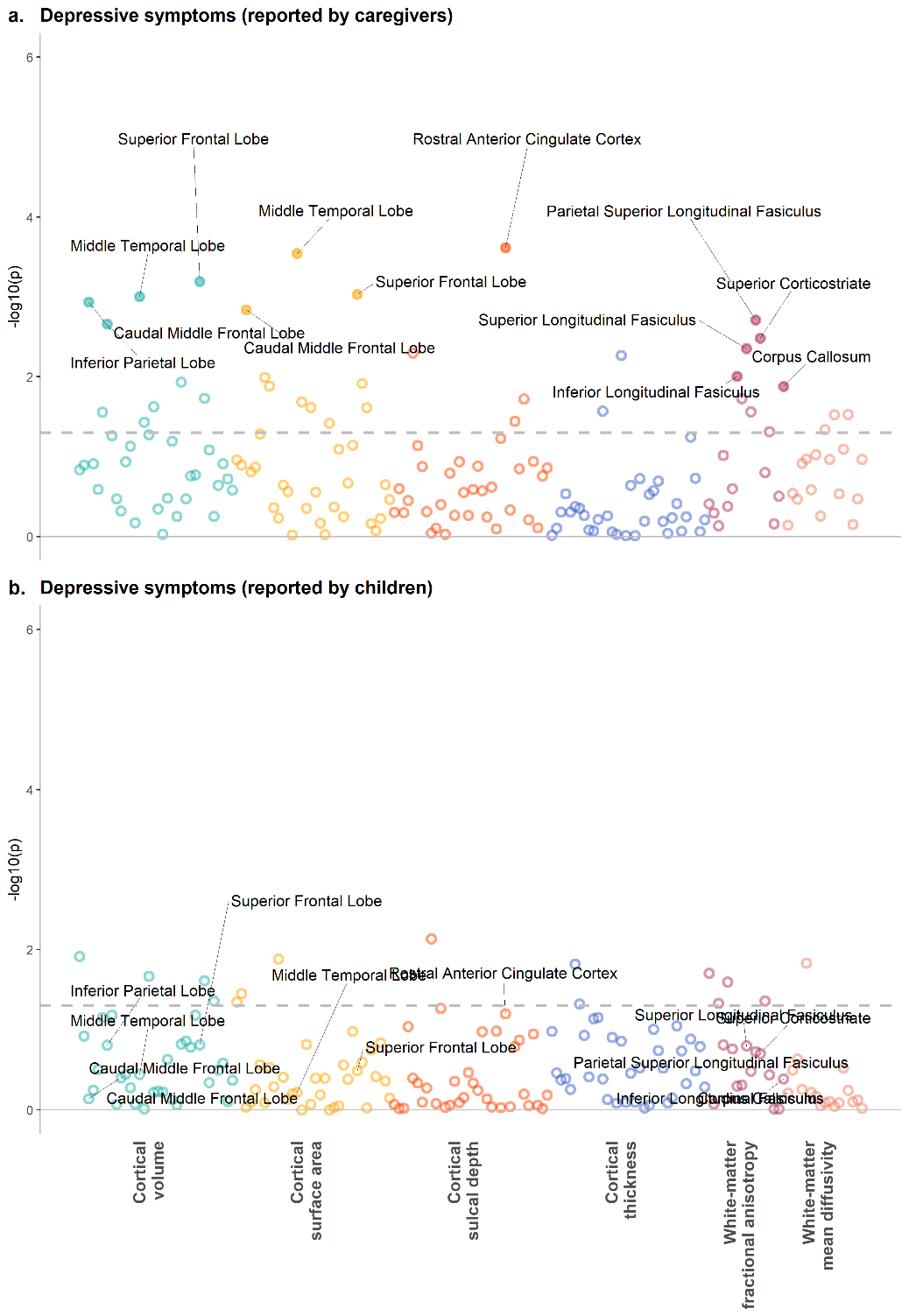


Figure S7. P-value plots for associations between MDD diagnosis and measures for single brain regions, controlling for ASR scale for severity of depression in caregivers. X axes represent measures for brain structural measures, and y axes represent -log10 transformed p-values. Panel (a) shows the results for depressive symptoms reported by caregivers on children, and panel (b) shows the results for symptoms reported by children themselves. Solid dots represent variables associated with depressive symptoms after FDR-correction. For clarity, threshold for significance after FDR-correction is shown as the pink dashed lines.


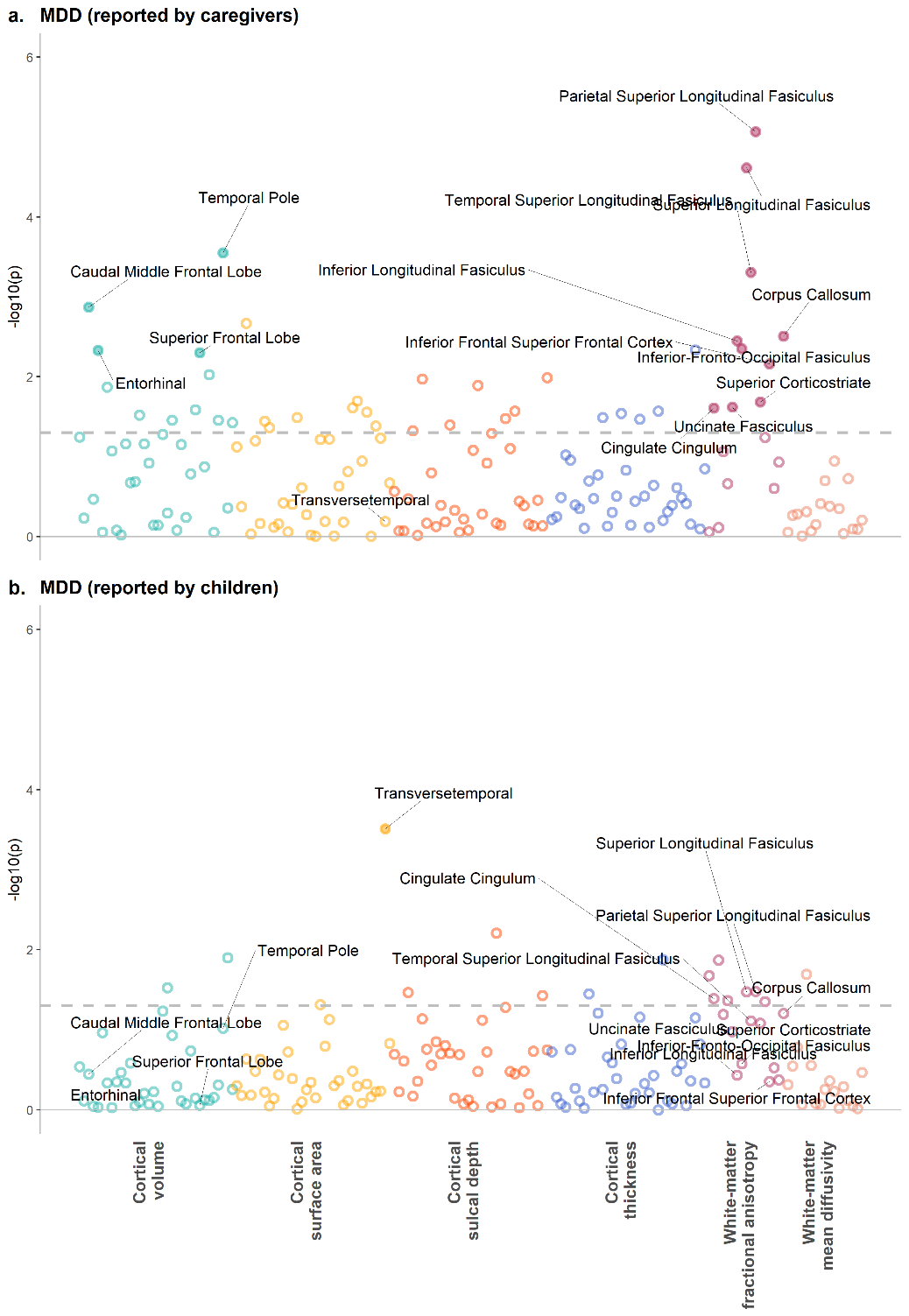


Figure S8. Correlations of effect sizes and p-values between the main analysis and the analysis controlling for ASR scale for severity of depression in caregivers (Supplementary Methods). X-axes represent statistics of the main model and the Y-axes represent statistics of controlling for severity of depression in caregivers. The left panel shows the correlation of standardised effect sizes (regression coefficient/Cohen’s d depending on which independent variable was used – MDD/depressive symptoms), and the right panel shows the correlation of p-values. In the right panel, the grey dashed line shows the threshold of nominal significance (p < 0.05).


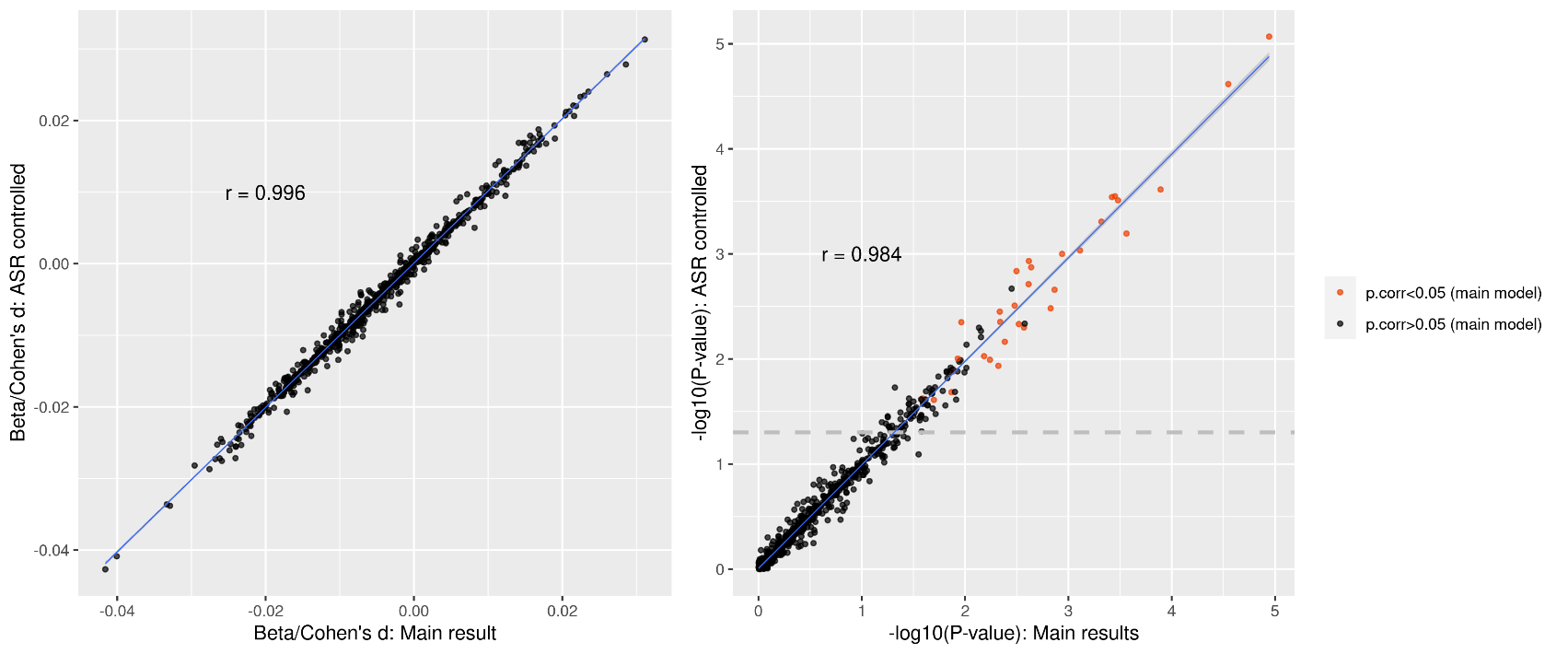


Figure S9. Leave-one-out analysis testing the association between general brain measures and depressive symptoms reported by caregivers. The x-axes represent standardised effect sizes. The y-axes represent the analysis leaving the given site out. The green dots represent the effect sizes by using the whole sample. The error bars represent 95% confidence intervals.


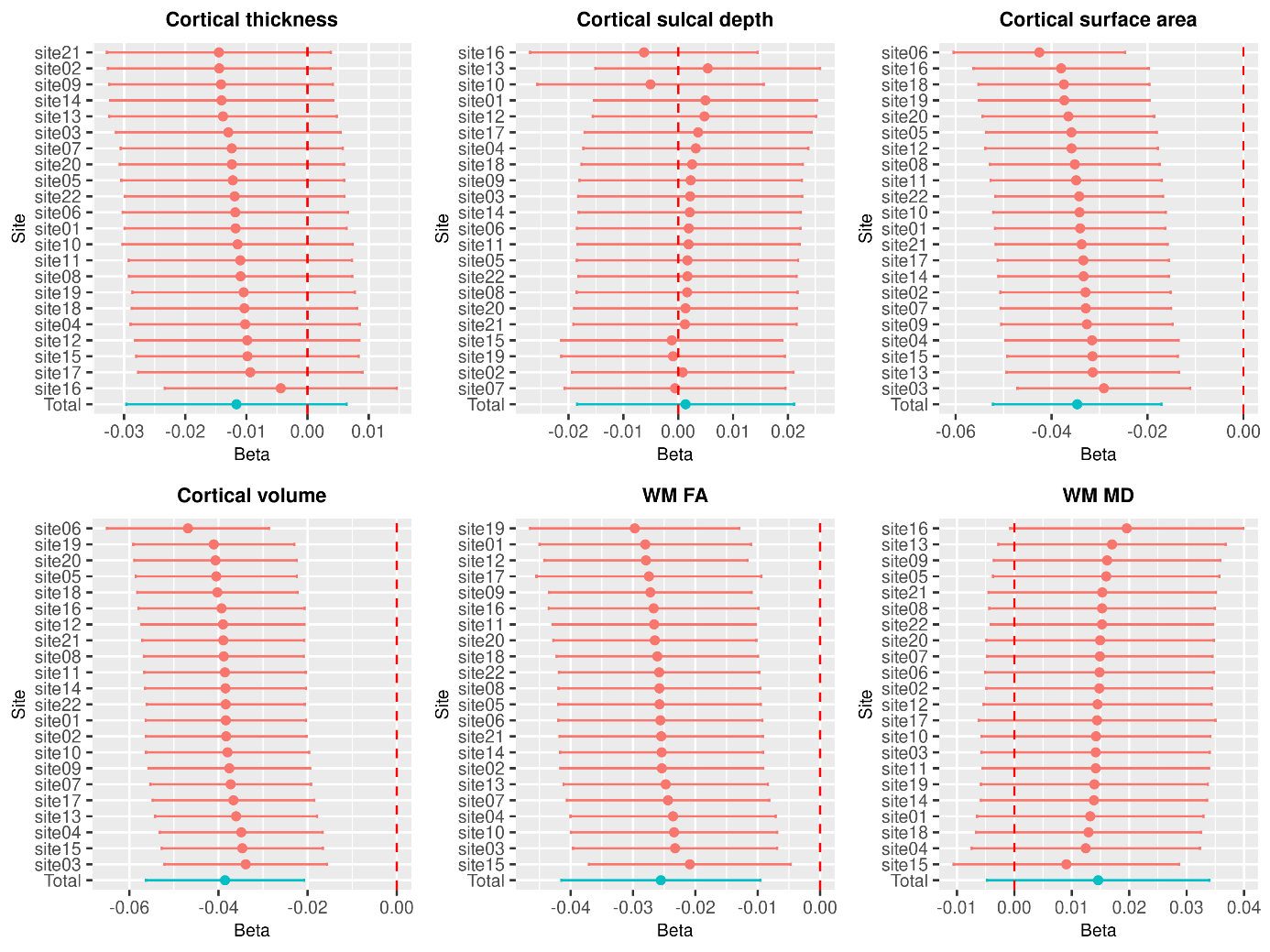


Figure S10. Leave-one-out analysis testing the association between general brain measures and depressive symptoms reported by children. The x-axes represent standardised effect sizes. The y-axes represent the analysis leaving the given site out. The green dots represent the effect sizes by using the whole sample. The error bars represent 95% confidence intervals.


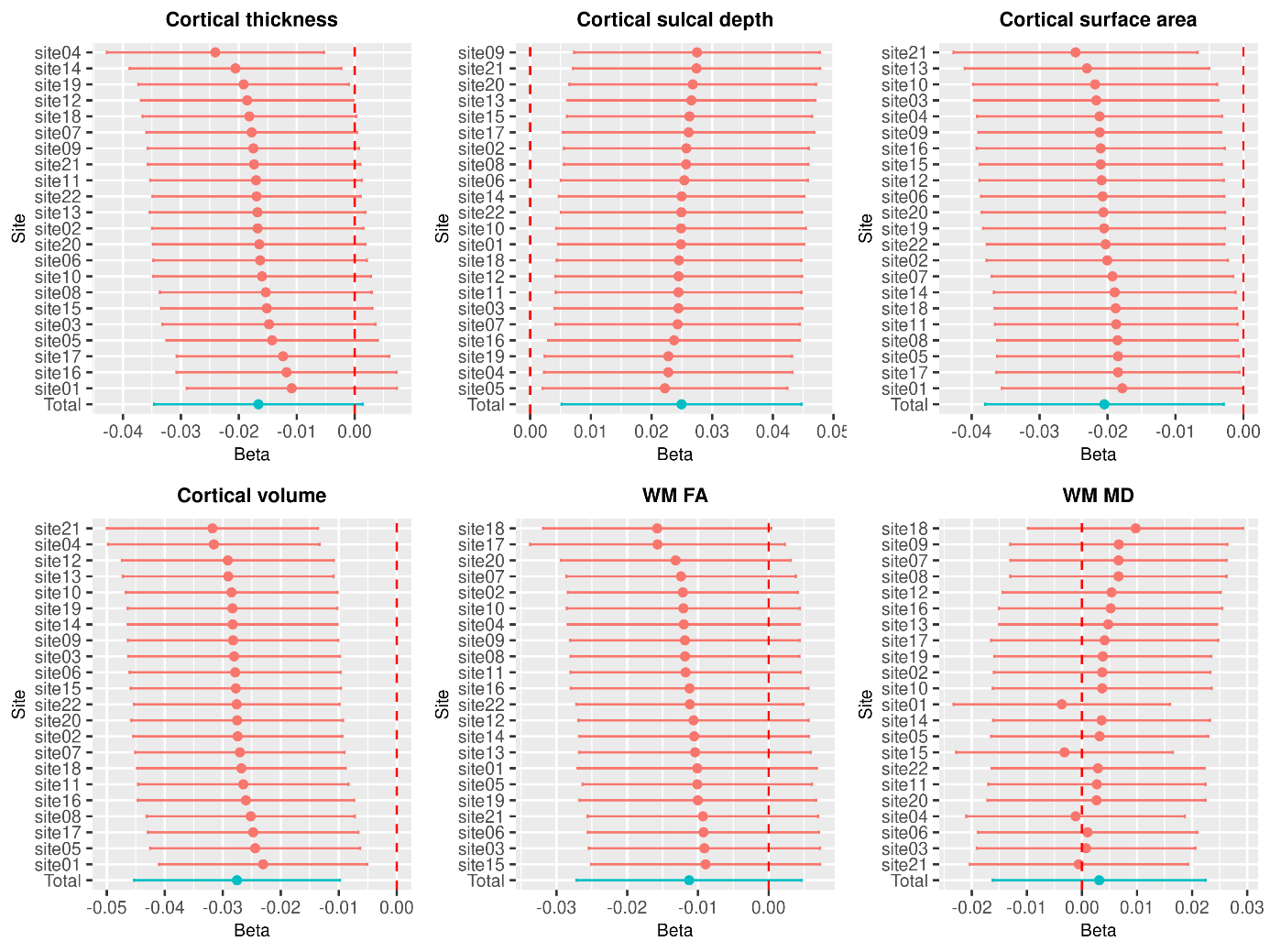


Figure S11. Leave-one-out analysis testing the association between general brain measures and MDD diagnosis reported by caregivers. The x-axes represent standardised effect sizes. The y-axes represent the analysis leaving the given site out. The green dots represent the effect sizes by using the whole sample. The error bars represent 95% confidence intervals.


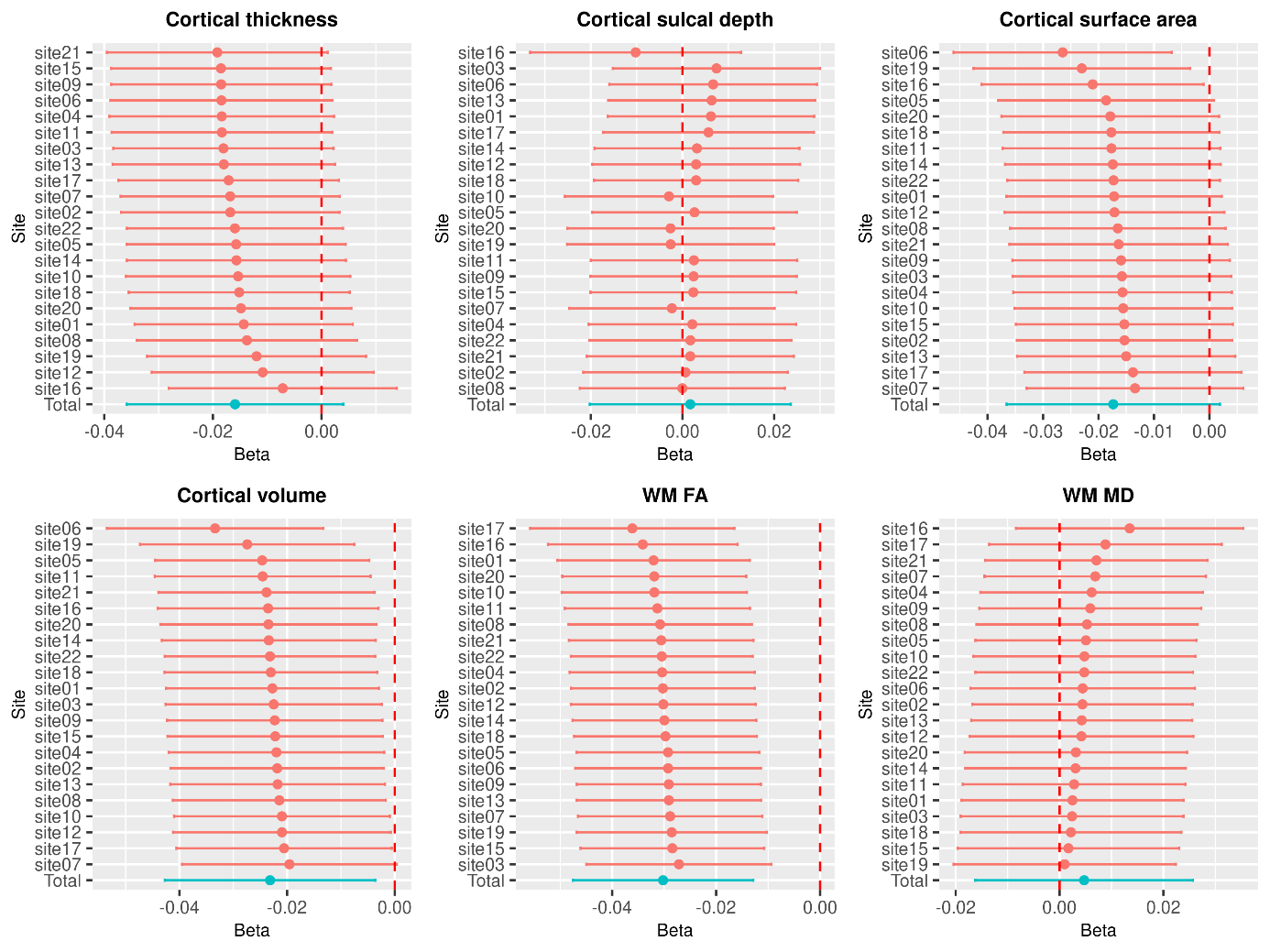


Figure S12. Leave-one-out analysis testing the association between general brain measures and MDD diagnosis reported by children. The x-axes represent standardised effect sizes. The y-axes represent the analysis leaving the given site out. The green dots represent the effect sizes by using the whole sample. The error bars represent 95% confidence intervals.


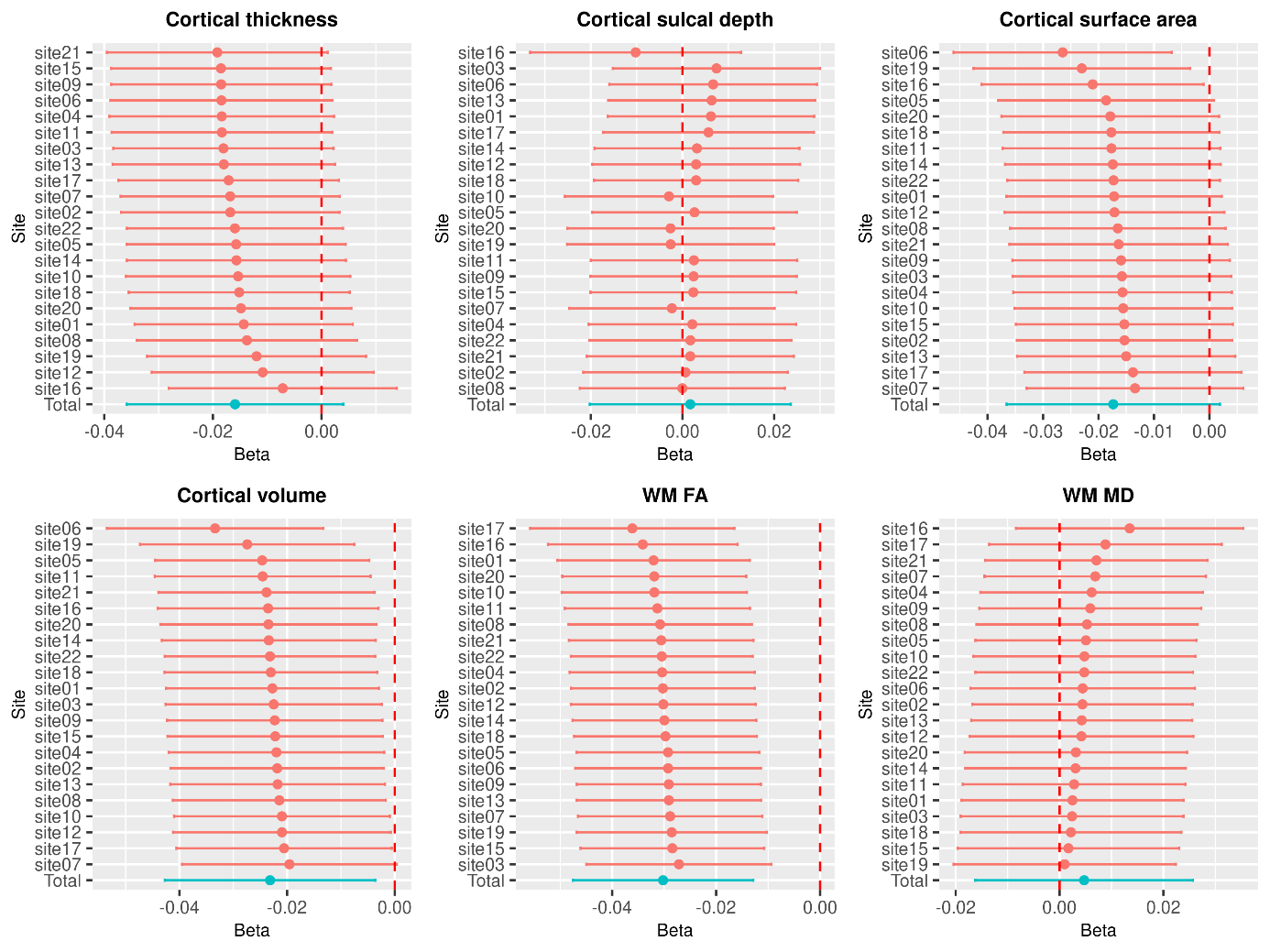


Figure S13. Associations between MDD, depressive symptoms and general measures of brain structures, controlling for MRI manufacturer. X-axes represent standardised effect sizes with error bars represent 95% confidence intervals, and y-axes represent each general measure of brain structure. Panel a shows the results for MDD/depressive symptoms reported by caregivers on children, and panel b shows the results for symptoms reported by children themselves.


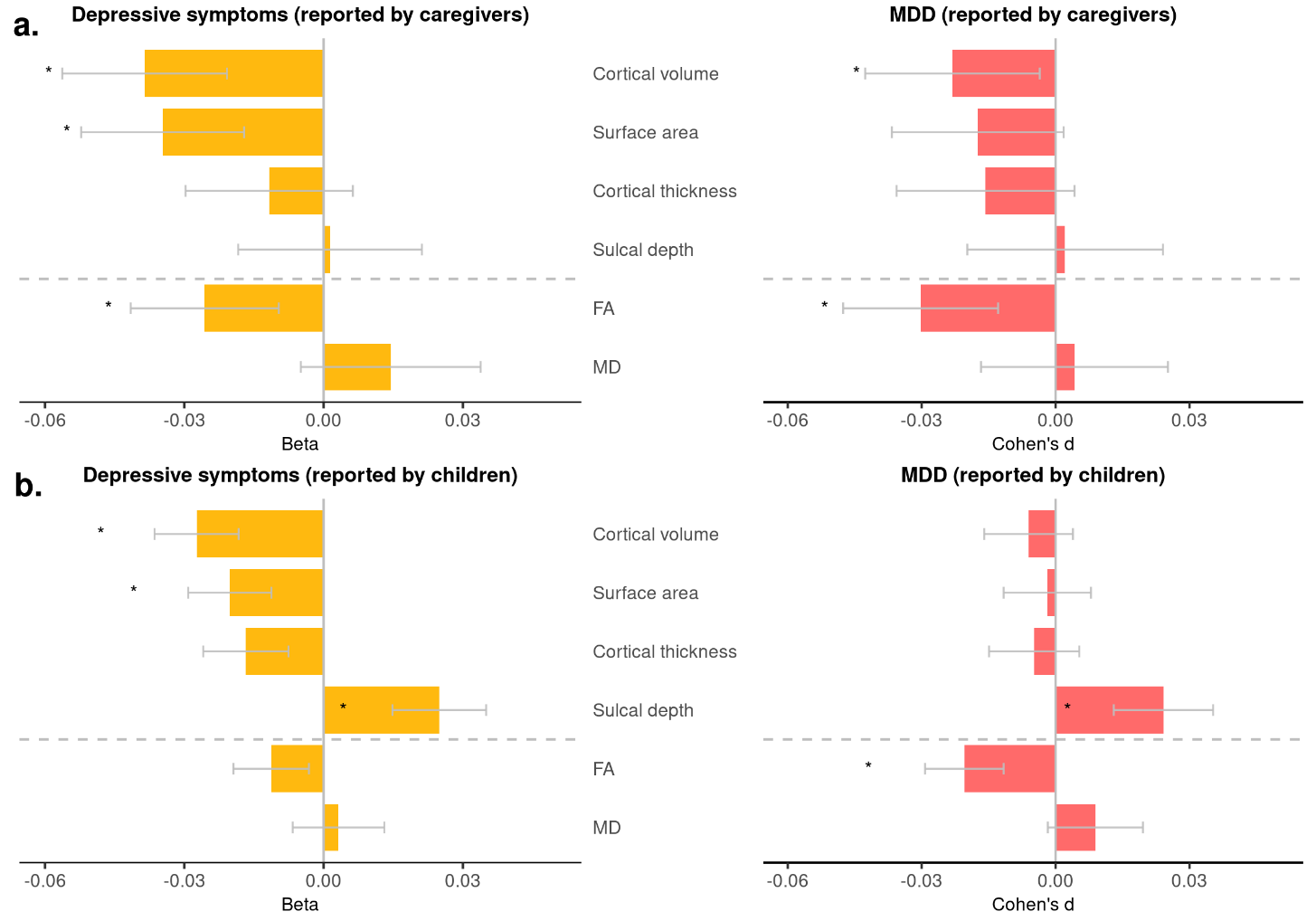


Figure S14. Correlations of effect sizes and p-values between the main model (not controlling for MRI manufacturer) and the secondary model controlling for MRI manufacturer. X-axes represent statistics of the main model and the Y-axes represent statistics of the secondary model. The left panel shows the correlation of standardised effect sizes (regression coefficient/Cohen’s d depending on which independent variable was used – MDD/depressive symptoms), and the right panel shows the correlation of p-values. In the right panel, the grey dashed line shows the threshold of nominal significance (p < 0.05).


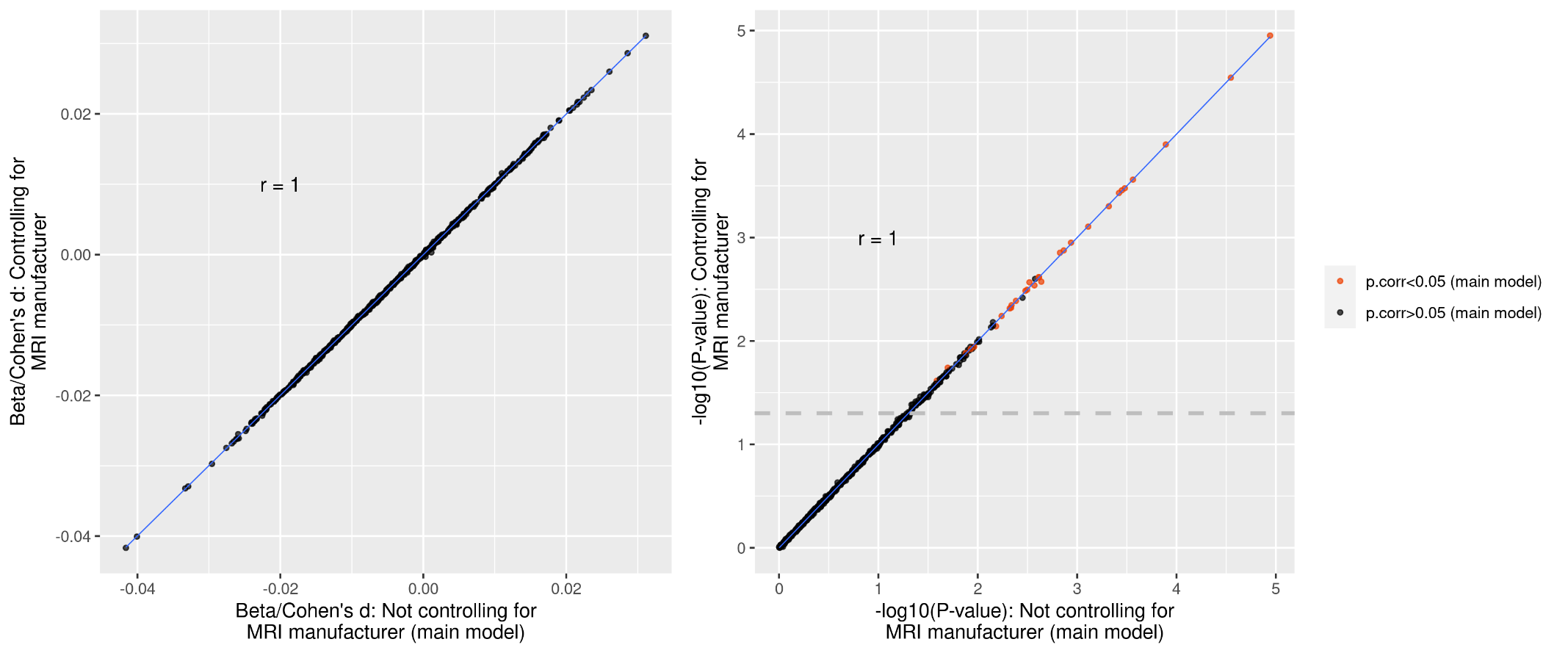


Figure S15. Associations between MDD, depressive symptoms and general measures of brain structures, controlling for medication. X-axes represent standardised effect sizes with error bars representing 95% confidence intervals, and y-axes represent each general measure of brain structure. Panel a shows the results for MDD/depressive symptoms reported by caregivers on children, and panel b shows the results for symptoms reported by children themselves.


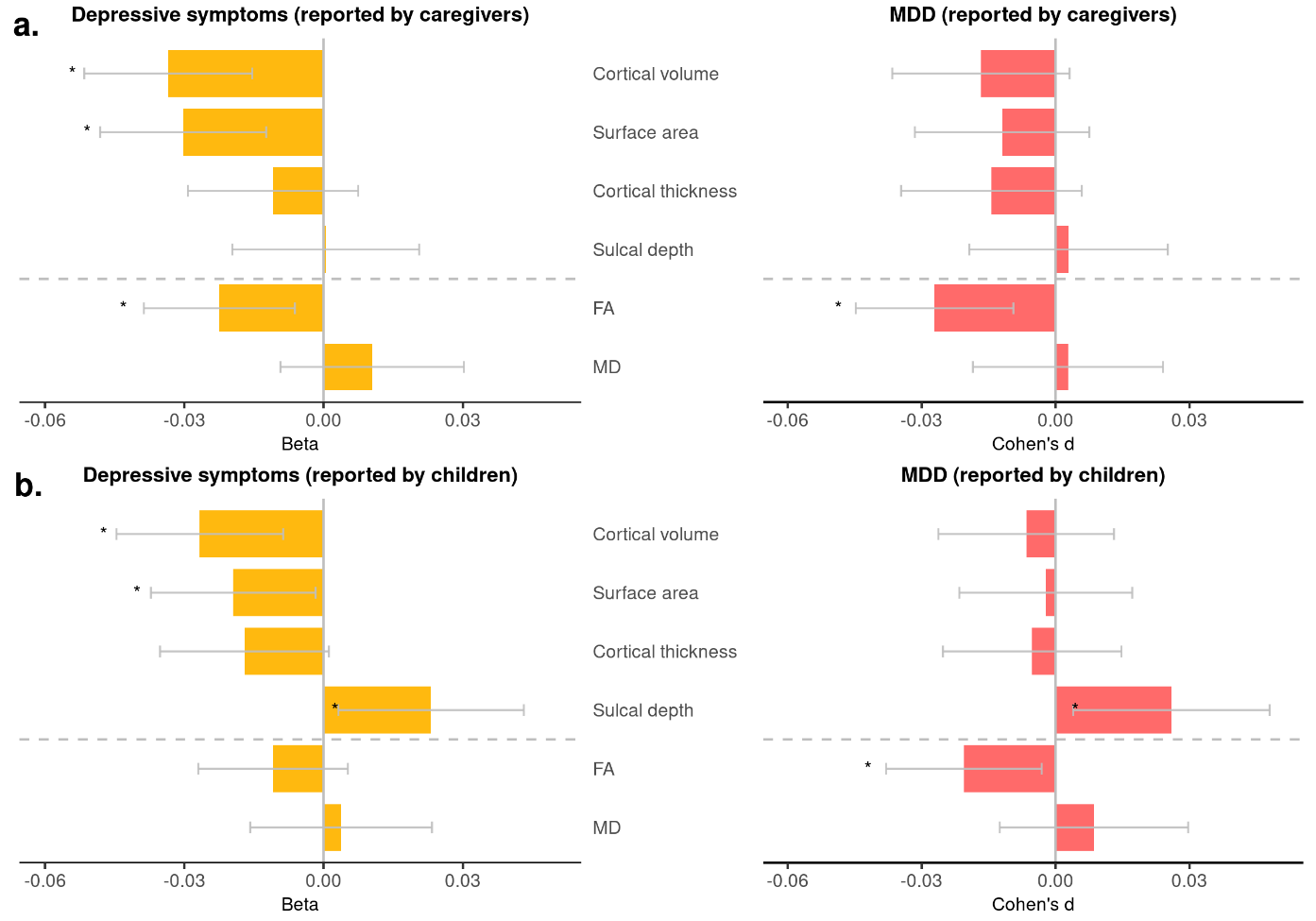


Figure S16. Correlations of effect sizes and p-values between the main model (not controlling for medication) and the secondary model controlling for medication. X-axes represent statistics of the main model and the Y-axes represent statistics of the secondary model. The left panel shows the correlation of standardised effect sizes (regression coefficient/Cohen’s d depending on which independent variable was used – MDD/depressive symptoms), and the right panel shows the correlation of p-values. In the right panel, the grey dashed line shows the threshold of nominal significance (p < 0.05).


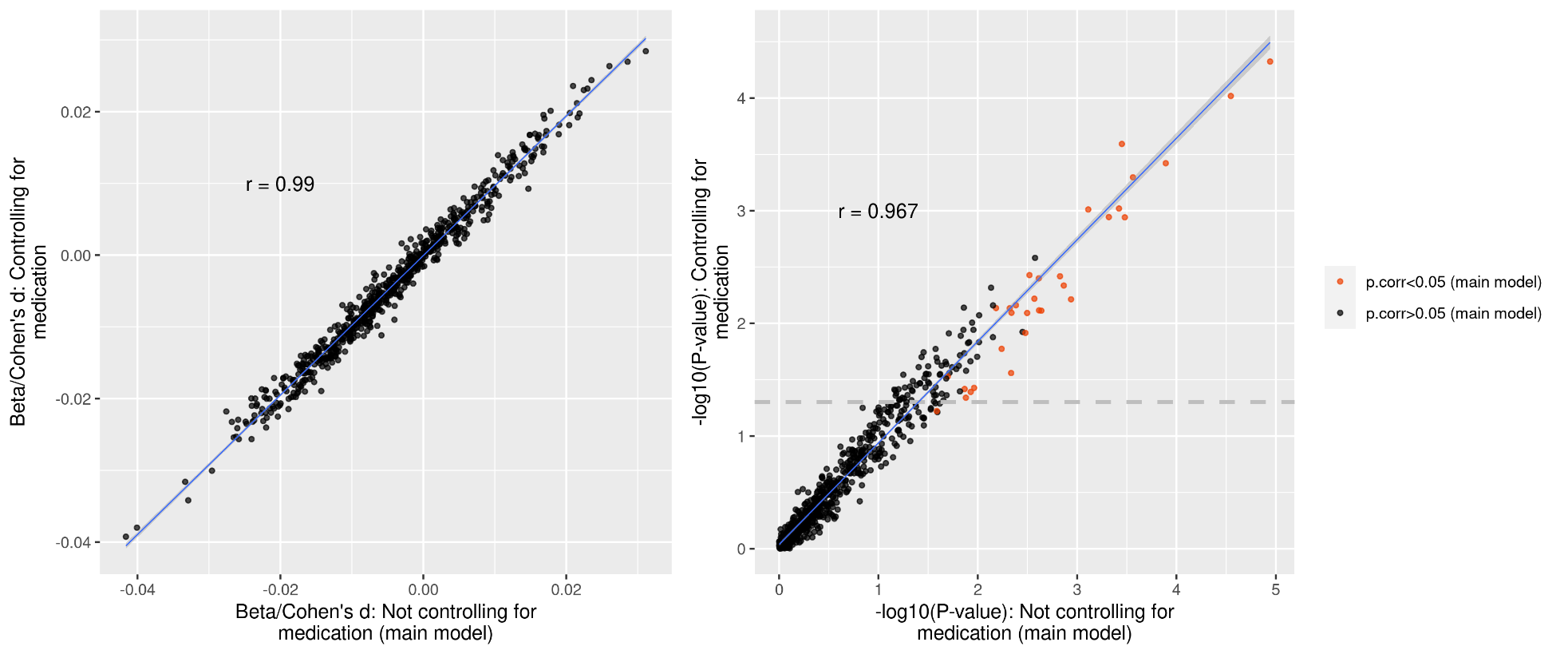


Figure S17. Associations between the average rating between caregiver and child reports of depressive symptoms and general measures of brain structures. X-axes represent standardised effect sizes, and y-axes represent each general measure of brain structure. Error bars represent 95% confidence intervals. Significant associations are highlighted with an asterisk.


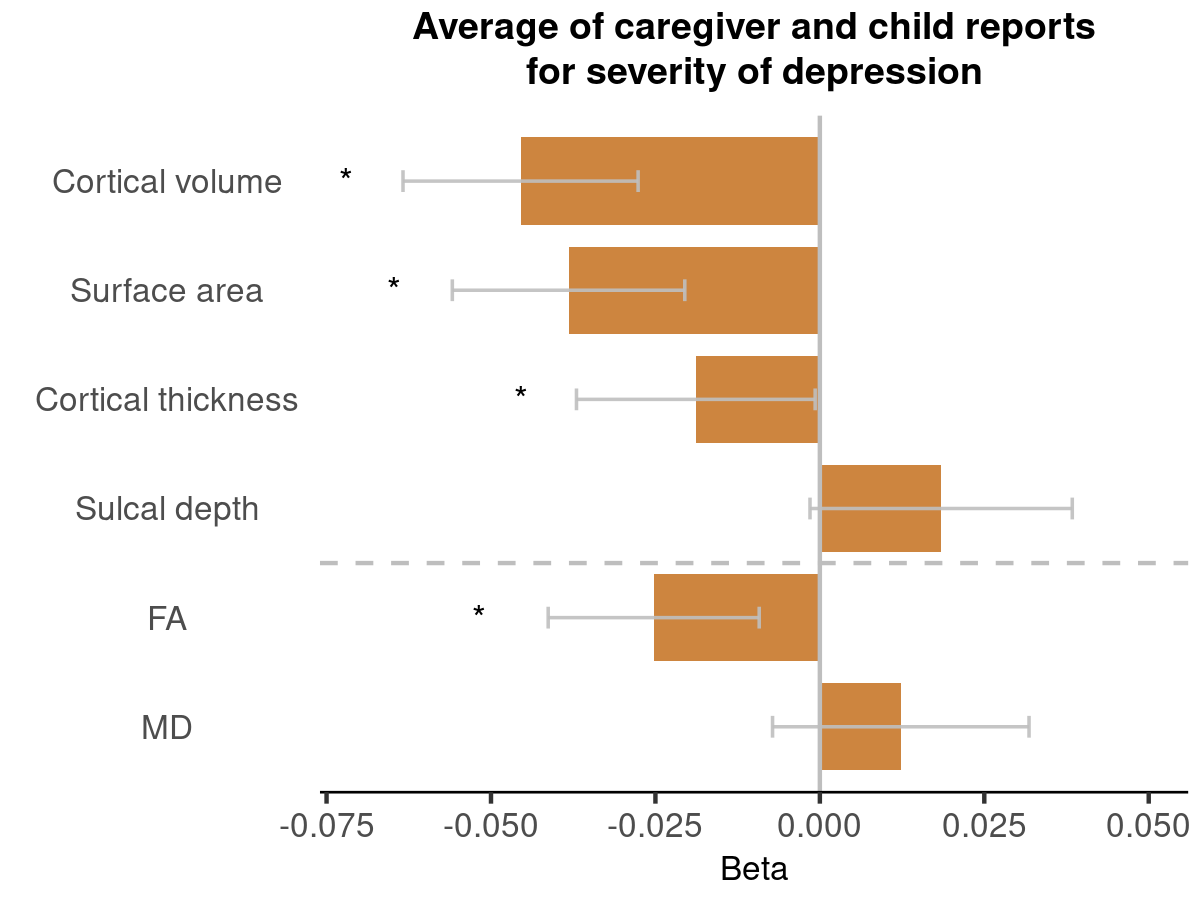


Figure S18. P-value plots for associations between average of caregiver and child report of severity of MDD and measures for single brain regions. X axes represent measures for brain structural measures, and y axes represent -log10 transformed p-values. Solid dots represent significant associations after FDR-correction. Pink dashed line represents nominally significance threshold. Those regions with the label ‘(+)’ were new associations found with average DS but not with parent-reported DS. Regions with the label ‘(-)’ were associations that were found with parent-reported DS but not with average DS.


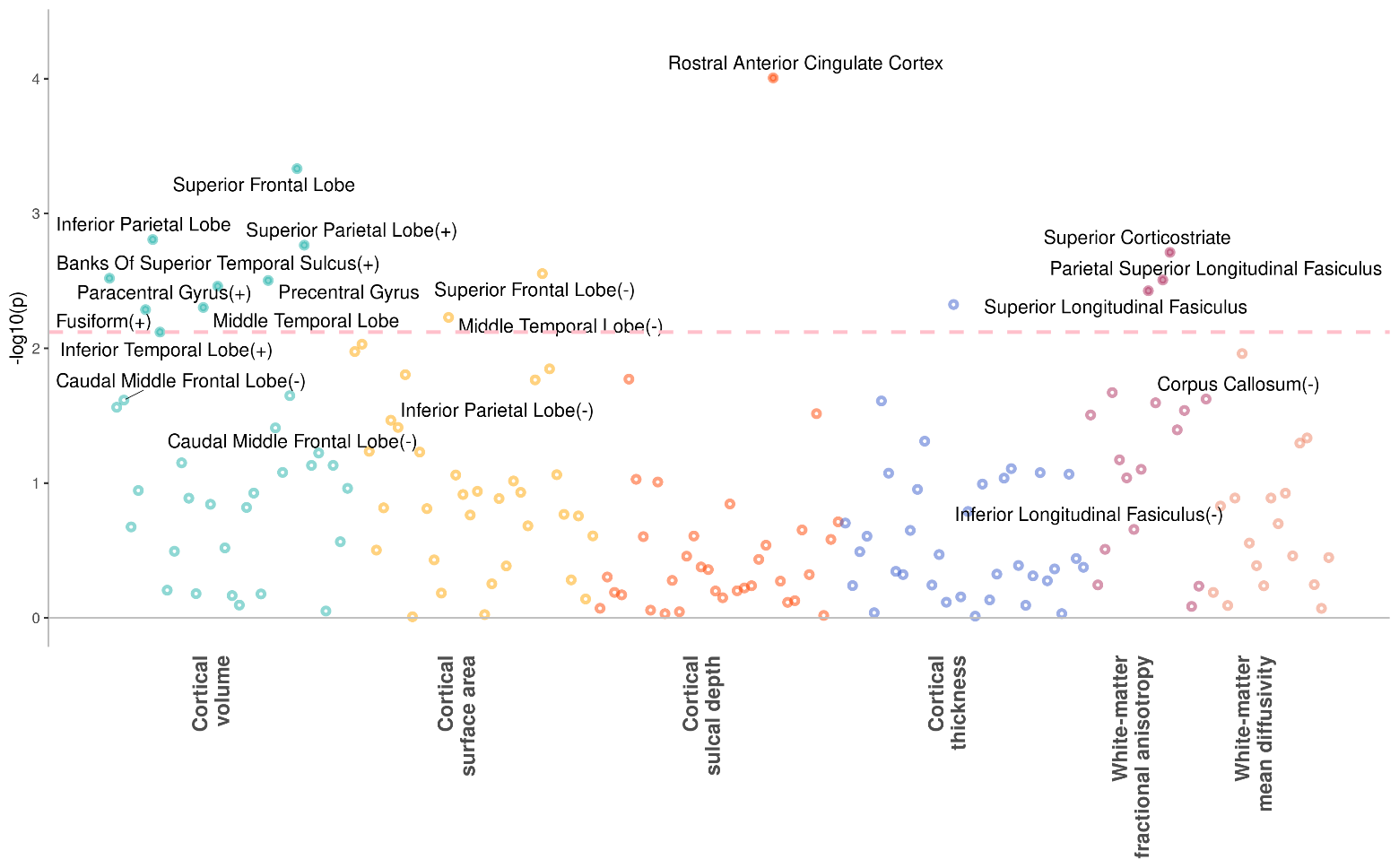


Figure S19. Associations between average of child and caregiver reports of depressive symptoms and regional measures of brain structures. Regional brain measures that were found significantly associated with caregiver report of depressive symptoms were chosen for this analysis and thus multiple comparison correction was conducted withing the tested associations only. X-axes represent standardised effect sizes, and y-axes represent each regional brain structure. Error bars represent 95% confidence intervals. Significant associations are highlighted with an asterisk.


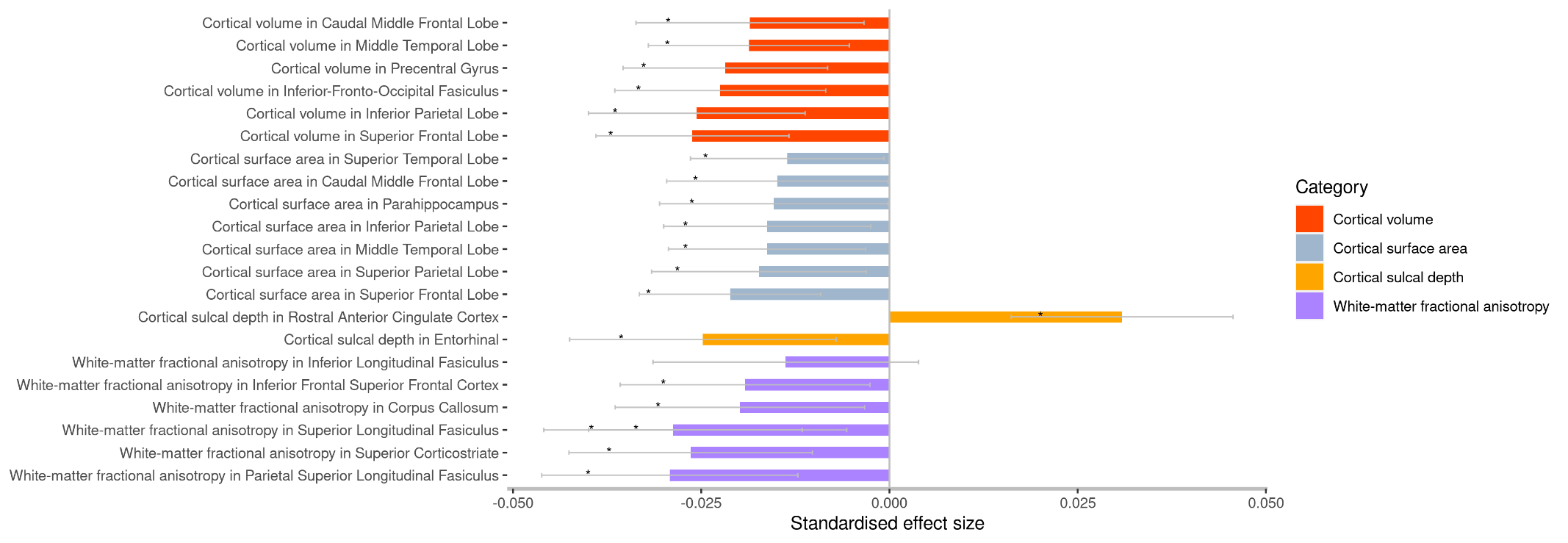


Figure S20. Reporter difference for each KSADS item. The X-axis represents the proportion of types of reporter comparisons (Caregiver > Child: caregiver=1 and child=0; Child > Caregiver: child=1 and caregiver=0; Caregiver = Child: caregiver=child=1 or caregiver=child=0). The y-axis represents each KSADS item used for deriving severity of MDD.


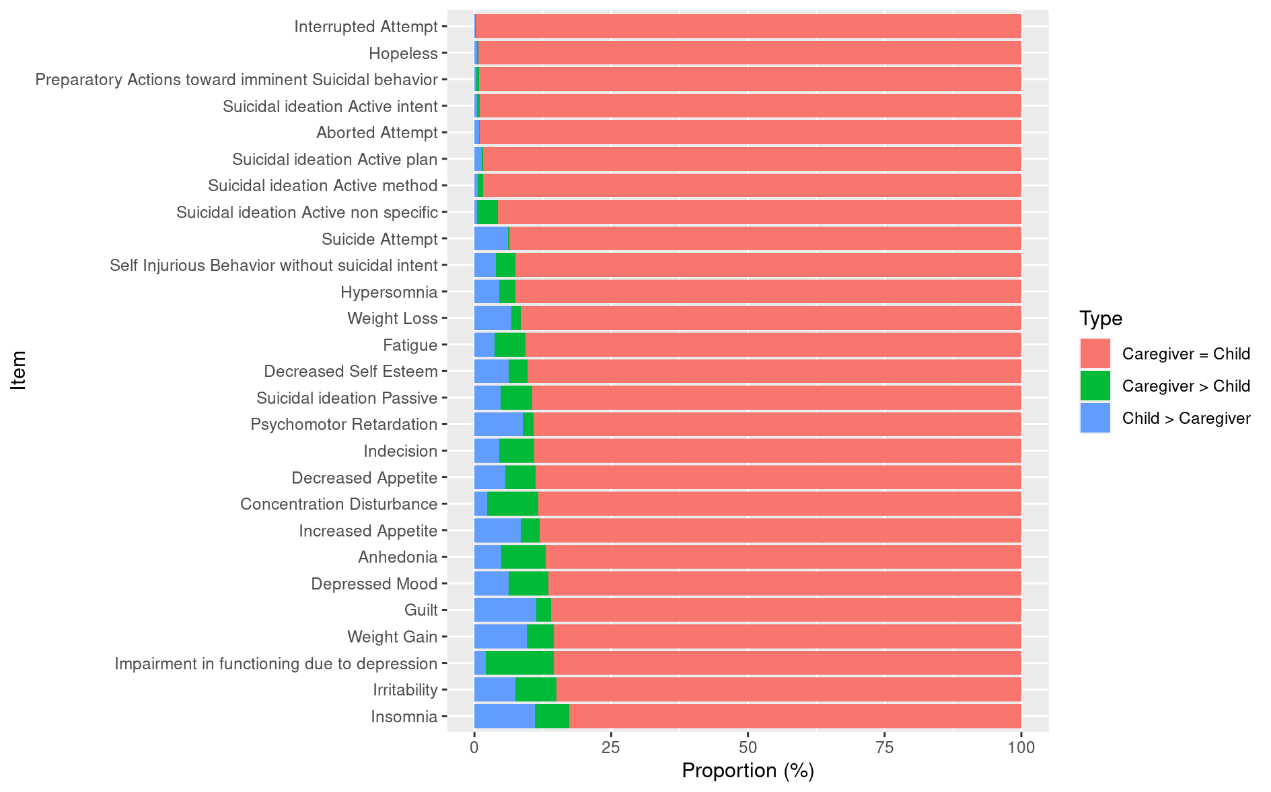


Figure S21. Correlations of effect sizes and p-values of using the subsample (N=3733) with post-processing QC data available and for those who passed QC in this subsample (N=3509). X-axes represent statistics of the main model and the Y-axes represent statistics of the secondary model. The left panel shows the correlation of standardised effect sizes (regression coefficient/Cohen’s d depending on which independent variable was used – MDD/depressive symptoms), and the right panel shows the correlation of p-values. In the right panel, the grey dashed line shows the threshold of nominal significance (p < 0.05).


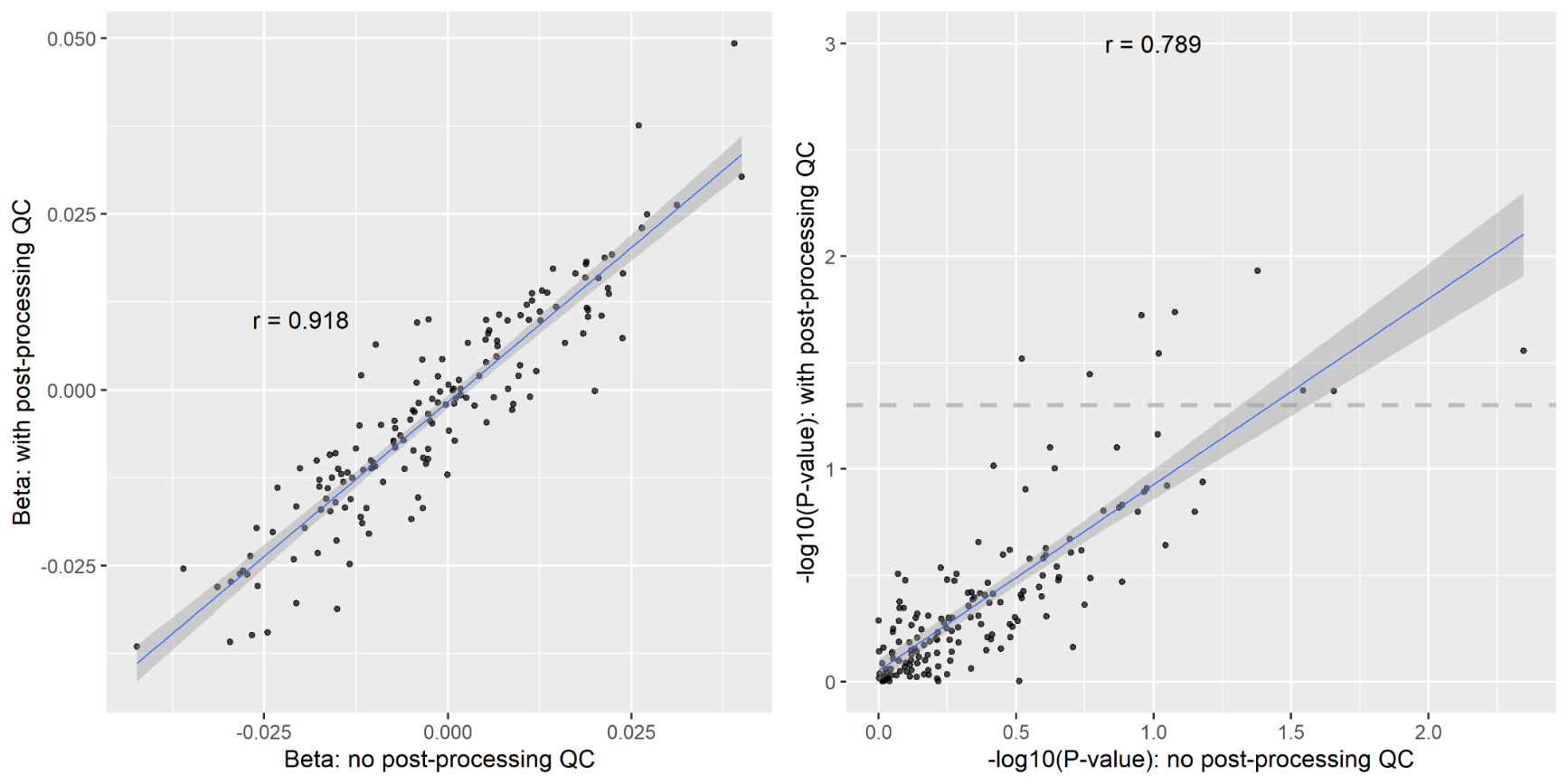


Figure S22. Associations between the absolute difference between caregiver and child reports of depressive symptoms and general measures of brain structures. X-axes represent standardised effect sizes, and y-axes represent each general measure of brain structure. Error bars represent 95% confidence intervals. Significant associations are highlighted with an asterisk.


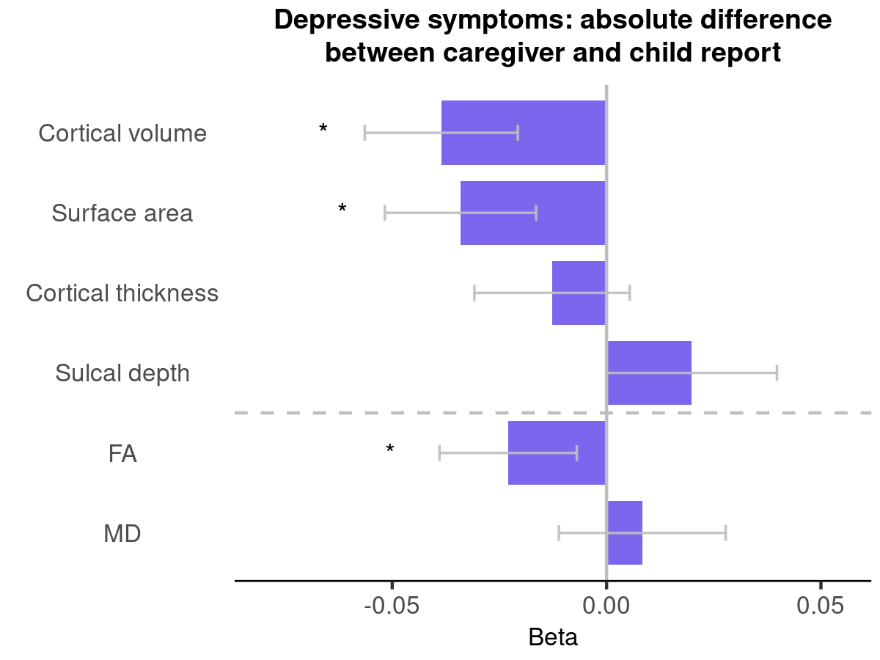


Figure S23. Associations between the absolute difference between child and caregiver reports of depressive symptoms regional measures of brain structures. Regional brain measures that were found significantly associated with caregiver report of depressive symptoms were chosen for this analysis. X-axes represent standardised effect sizes, and y-axes represent each regional brain structure. Error bars represent 95% confidence intervals. Significant associations are highlighted with an asterisk.


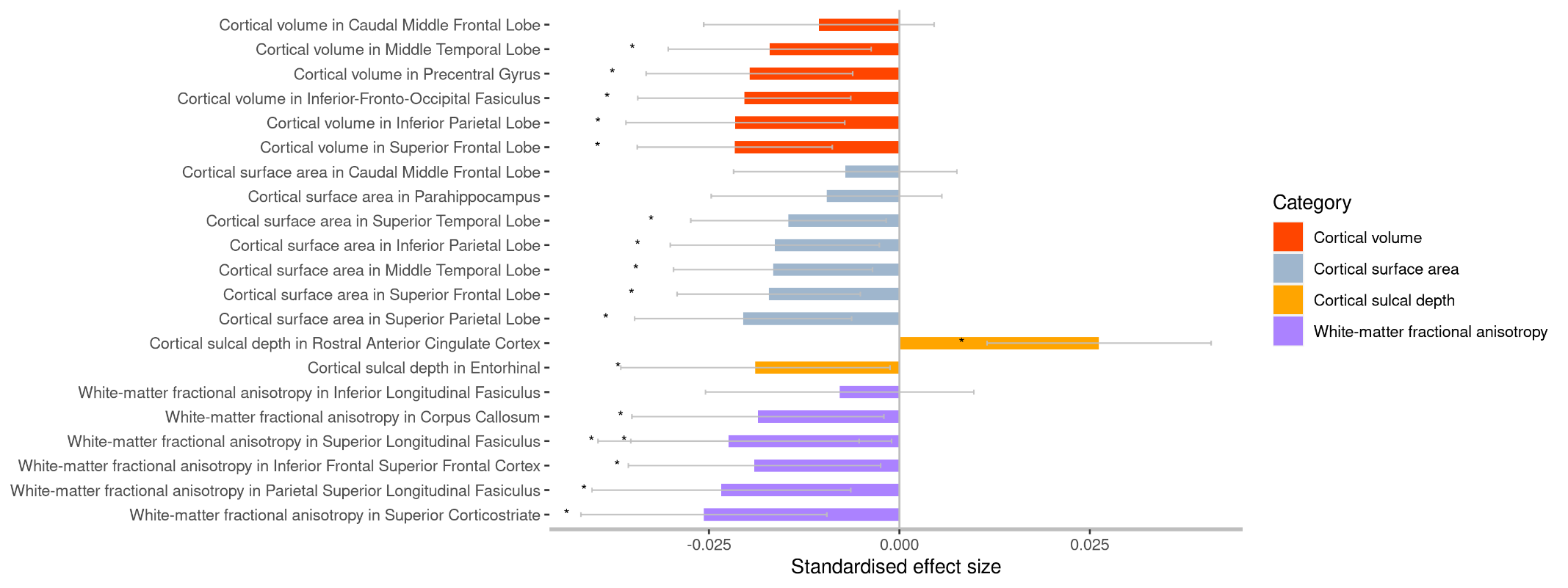


Figure S24. Distribution of CBCL-measured DS under each MDD severity category classified using KSADS (both KSADS and CBCL measures were reported by caregivers). X axis represents CBCL-measured DS. Each row’s y axis represents distribution density. The vertical lines in each distribution represents the mean of the given distribution.


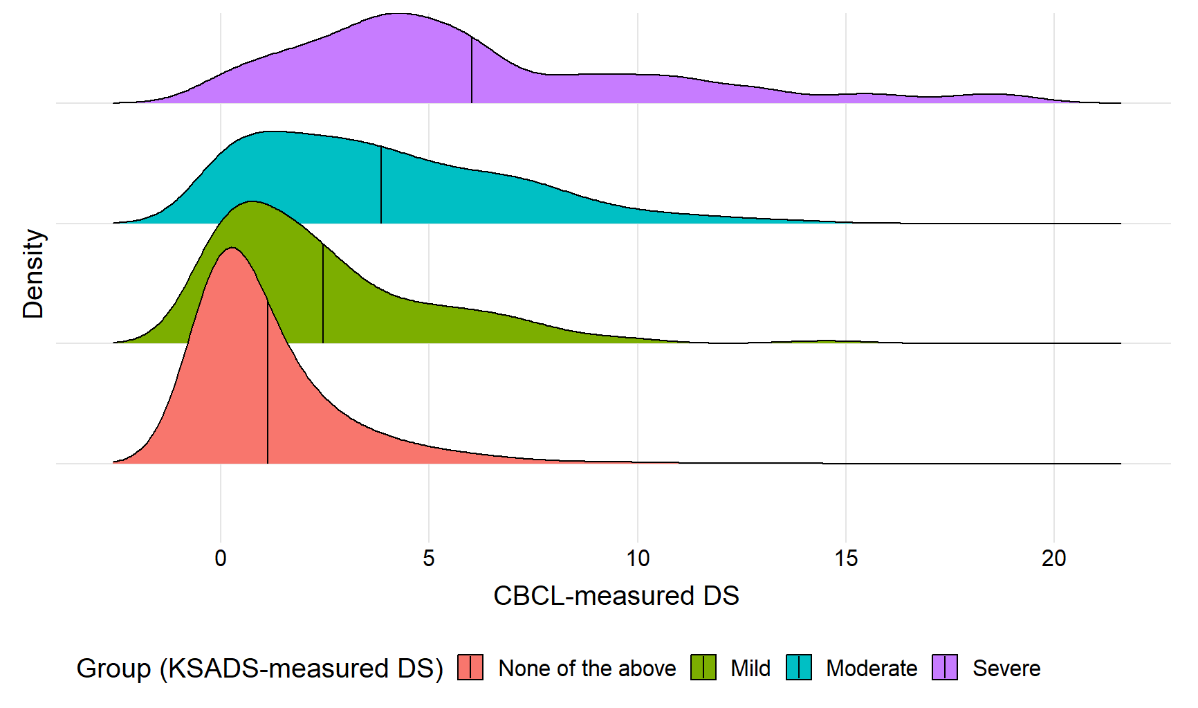


Figure S25. Associations between CBCL DSM-5-oriented score of depression and general measures of brain structures. X-axes represent standardised effect sizes, and y-axes represent each general measure of brain structure. Error bars represent 95% confidence intervals. Significant associations are highlighted with an asterisk.


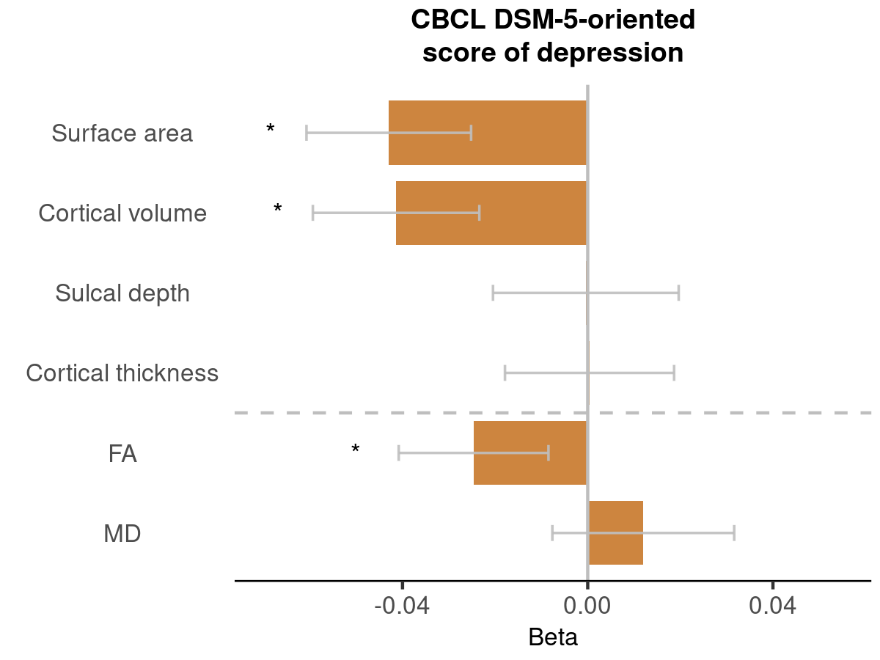


Figure S26. P-value plots for associations between depressive symptoms assessed by CBCL (the Child Behaviour Checklist, reported by caregivers) and measures for single brain regions. X axes represent measures for brain structural measures, and y axes represent -log10 transformed p-values. Panel (a) shows the results for depressive symptoms reported by caregivers on children, and panel (b) shows the results for symptoms reported by children themselves. Solid dots represent variables associated with depressive symptoms after FDR-correction. For clarity, threshold for significance after FDR-correction is shown as the pink dashed lines.


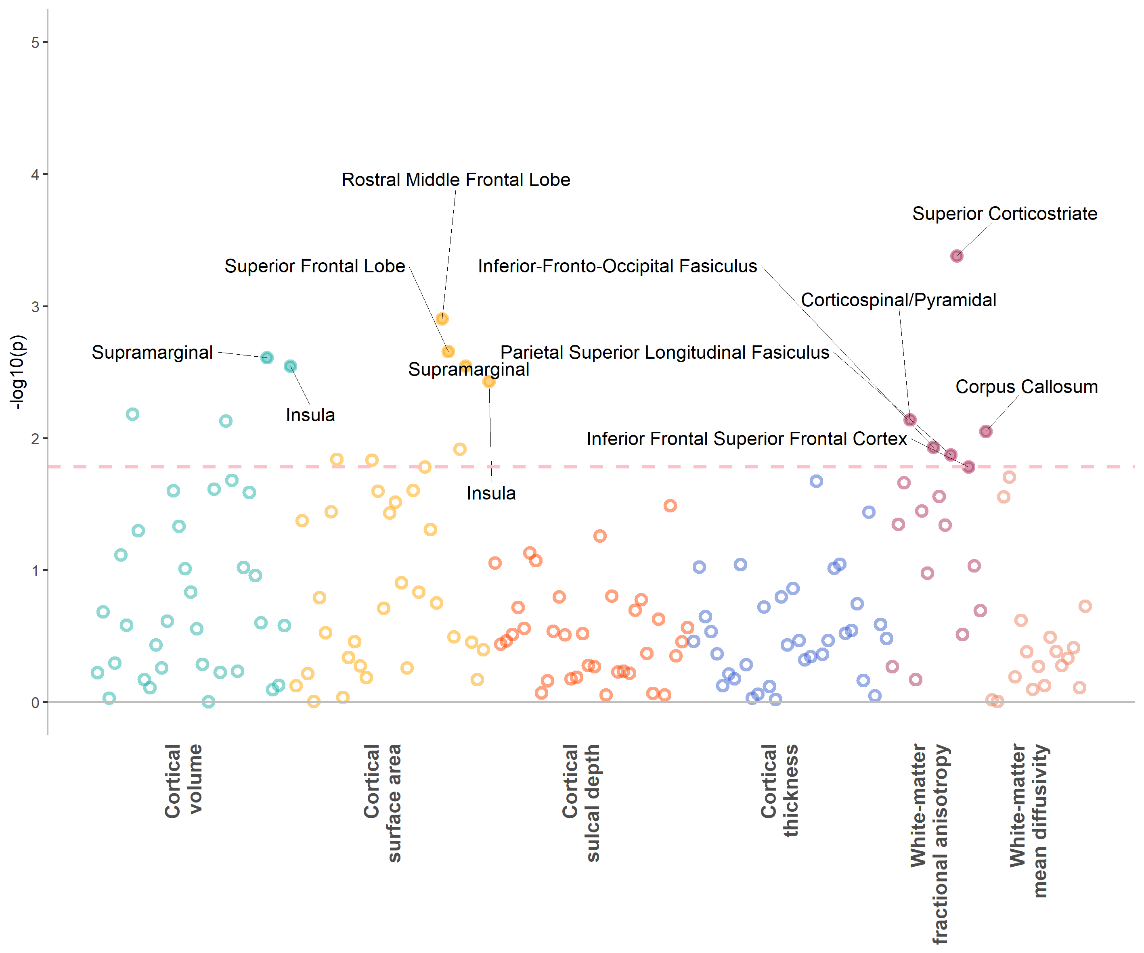


Figure S27. Associations between the caregiver report and general measures of brain structures after accounting for comorbidity of Bipolar I, Bipolar II, ADHD, Psychosis and Conduct disorder. X-axes represent standardised effect sizes, and y-axes represent each general measure of brain structure. Error bars represent 95% confidence intervals. Significant associations are highlighted with an asterisk.


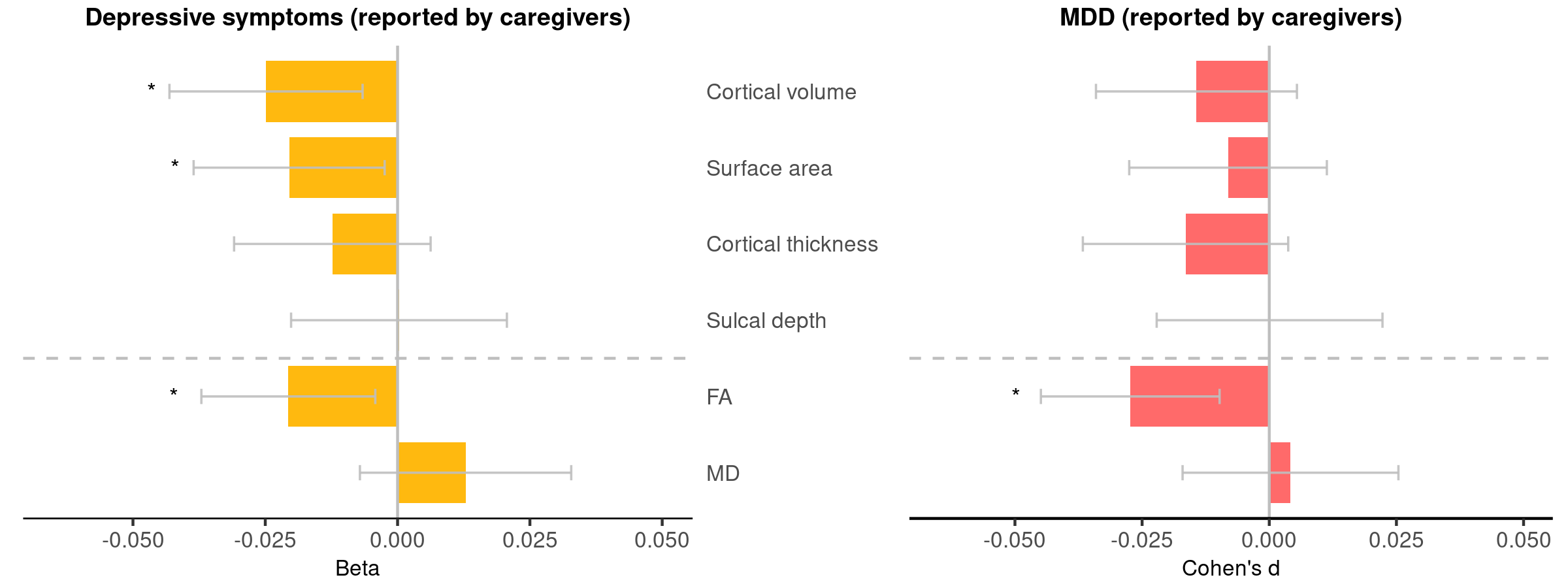


Figure S28. Correlations of effect sizes and p-values for the main model and a secondary model accounting for comorbidity of Bipolar I, Bipolar II, ADHD, Psychosis and Conduct disorder. X-axes represent statistics of the main model and the Y-axes represent statistics of the secondary model. The left panel shows the correlation of standardised effect sizes (regression coefficient/Cohen’s d depending on which independent variable was used – MDD/depressive symptoms), and the right panel shows the correlation of p-values. In the right panel, the grey dashed line shows the threshold of nominal significance (p < 0.05).


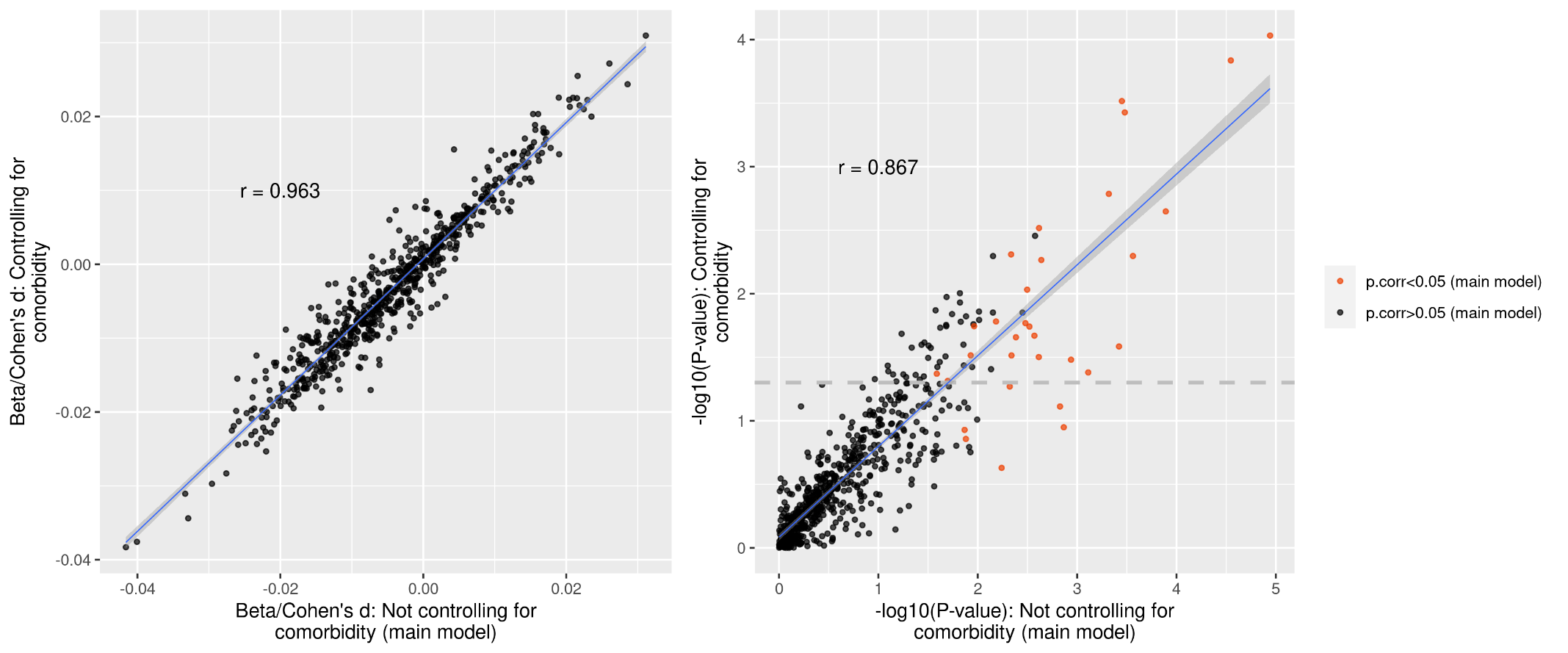


Supplementary Data 1. Results for associations between MDD/Depressive symptoms and global neuroimaging measures. MDD.caregiver = MDD based on reports by caregivers, Depressive symptoms.p = depressive symptoms based on reports by caregivers, MDD.child = MDD based on reports by children and Depressive symptoms.y = depressive symptoms based on reports by children.

Supplementary Data 2. Results for associations between MDD/Depressive symptoms and cortical thickness. MDD.caregiver = MDD based on reports by caregivers, Depressive symptoms.p = depressive symptoms based on reports by caregivers, MDD.child = MDD based on reports by children and Depressive symptoms.y = depressive symptoms based on reports by children.

Supplementary Data 3. Results for associations between MDD/Depressive symptoms and cortical surface area. MDD.caregiver = MDD based on reports by caregivers, Depressive symptoms.p = depressive symptoms based on reports by caregivers, MDD.child = MDD based on reports by children and Depressive symptoms.y = depressive symptoms based on reports by children.

Supplementary Data 4. Results for associations between MDD/Depressive symptoms and cortical sulcal depth. MDD.caregiver = MDD based on reports by caregivers, Depressive symptoms.p = depressive symptoms based on reports by caregivers, MDD.child = MDD based on reports by children and Depressive symptoms.y = depressive symptoms based on reports by children.

Supplementary Data 5. Results for associations between MDD/Depressive symptoms and cortical volume. MDD.caregiver = MDD based on reports by caregivers, Depressive symptoms.p = depressive symptoms based on reports by caregivers, MDD.child = MDD based on reports by children and Depressive symptoms.y = depressive symptoms based on reports by children.

Supplementary Data 6. Results for associations between MDD/Depressive symptoms and white-matter fractional anisotropy. MDD.caregiver = MDD based on reports by caregivers, Depressive symptoms.p = depressive symptoms based on reports by caregivers, MDD.child = MDD based on reports by children and Depressive symptoms.y = depressive symptoms based on reports by children.

Supplementary Data 7. Results for associations between MDD/Depressive symptoms and white-matter mean diffusivity. MDD.caregiver = MDD based on reports by caregivers, Depressive symptoms.p = depressive symptoms based on reports by caregivers, MDD.child = MDD based on reports by children and Depressive symptoms.y = depressive symptoms based on reports by children.
